# Evolutionary Dynamics of Human Rotaviruses: Balancing Reassortment with Preferred Genome Constellations

**DOI:** 10.1371/journal.ppat.1000634

**Published:** 2009-10-23

**Authors:** Sarah M. McDonald, Jelle Matthijnssens, John K. McAllen, Erin Hine, Larry Overton, Shiliang Wang, Philippe Lemey, Mark Zeller, Marc Van Ranst, David J. Spiro, John T. Patton

**Affiliations:** 1 Laboratory of Infectious Diseases, National Institute of Allergy and Infectious Diseases, National Institutes of Health, Bethesda, Maryland, United States of America; 2 Laboratory of Clinical and Epidemiological Virology, Department of Microbiology and Immunology, Rega Institute for Medical Research, K.U. Leuven, Leuven, Belgium; 3 The J. Craig Venter Institute, Rockville, Maryland, United States of America; Cornell University, United States of America

## Abstract

Group A human rotaviruses (RVs) are a major cause of severe gastroenteritis in infants and young children. Yet, aside from the genes encoding serotype antigens (VP7; G-type and VP4; P-type), little is known about the genetic make-up of emerging and endemic human RV strains. To gain insight into the diversity and evolution of RVs circulating at a single location over a period of time, we sequenced the eleven-segmented, double-stranded RNA genomes of fifty-one G3P[8] strains collected from 1974 to 1991 at Children's Hospital National Medical Center, Washington, D. C. During this period, G1P[8] strains typically dominated, comprising on average 56% of RV infections each year in hospitalized children. A notable exception was in the 1976 and 1991 winter seasons when the incidence of G1P[8] infections decreased dramatically, a trend that correlated with a significant increase in G3P[8] infections. Our sequence analysis indicates that the 1976 season was characterized by the presence of several genetically distinct, co-circulating clades of G3P[8] viruses, which contained minor but significant differences in their encoded proteins. These 1976 lineages did not readily exchange gene segments with each other, but instead remained stable over the course of the season. In contrast, the 1991 season contained a single major clade, whose genome constellation was similar to one of the 1976 clades. The 1991 clade may have gained a fitness advantage after reassorting with as of yet unidentified RV strain(s). This study reveals for the first time that genetically distinct RV clades of the same G/P-type can co-circulate and cause disease. The findings from this study also suggest that, although gene segment exchange occurs, most reassortant strains are replaced over time by lineages with preferred genome constellations. Elucidation of the selective pressures that favor maintenance of RVs with certain sets of genes may be necessary to anticipate future vaccine needs.

## Author Summary

Rotaviruses are the most important cause of severe diarrhea in infants and young children. Due to the segmented nature of their genomes, rotaviruses can exchange (reassort) genes during co-infections, a feature that is predicted to generate new, possibly more dangerous virus strains. However, the amount of gene reassortment occurring in nature is not known, as very few rotavirus genomes have been sequenced. To better understand the genetic make-up of rotaviruses circulating at a single location over a period of time, we sequenced the genomes of fifty-one isolates recovered from sick children from 1974 to 1991 at Children's Hospital National Medical Center, Washington, D. C. By analyzing these sequences, we found that several distinct groups (clades) of rotaviruses co-circulated and caused disease in a single epidemic season. In contrast to what was previously thought, very few rotaviruses exchanged gene segments with each other; instead, the genome constellations of the viruses remained relatively stable. We also discovered that these distinct rotavirus clades encode different viral proteins, which may be important in the development of effective vaccines. Together, the findings from this first large-scale rotavirus genomics project provide unparalleled insight into how these pathogens evolve during their spread through the human population.

## Introduction

Group A rotaviruses (RVs) are the most important etiological agents of acute, dehydrating gastroenteritis in young children. It is estimated that RV infections result in more than 500,000 deaths each year worldwide, the vast majority of which occur in developing countries [1],[2]. These pathogens are transmitted via the fecal-oral route with peak disease frequency occurring in cooler, winter months [3]. The infectious RV virion is a non-enveloped, triple-layered particle that encapsidates an eleven-segmented, double-stranded (ds) RNA genome [4]. Cumulatively, the viral genome encodes five or six nonstructural proteins (NSP1-5, and sometimes, NSP6) and six capsid proteins (VP1-4, VP6, and VP7) [3]. The outermost capsid proteins, VP7 and VP4, induce neutralizing antibodies during infection of a host; serotypes defined by these proteins are a traditional method of characterizing RV isolates [3]. Based on the sequences of genes encoding the serotype antigens, strains have been more recently classified into G-types (VP7) and P-types (VP4) [3],[5]. To date, a total of 22 G-type and 31 P-type strains have been described in the literature [6],[7],[8],[9],[10].

Ongoing epidemiological surveillance indicates that strains with G-types of G1, G2, G3, and G4, and those with P-types of P[4] and P[8], are the most prevalent cause of RV disease in humans. As such, these G/P-types are included in the currently licensed RV vaccines (RotaTeq and Rotarix) [11]. However, RV G/P-type distribution varies considerably from year-to-year for reasons that are not well understood [12]. For instance, an increased occurrence of one G/P-type will often accompany decreases in other G/P-types during a specific epidemic season [12],[13]. This phenomenon suggests that an emergent G/P-type may gain an advantage due to transient, but as of yet unidentified, selective pressure(s). In addition to the annual fluctuation in G/P-type distribution, the incidence of unusual types, such as G5, G6, G8, G9, G12, and P[6] is rising in certain geographical locations [11],[14]. Despite the observation that RV G/P-type diversity seems to be an ever-changing landscape, little is known about the genetic make-up of circulating strains. Compared to the wealth of sequence information for serotype antigens VP7 and VP4, few sequences are available for genes encoding other viral proteins. Studies of segmented RNA viruses, such as influenza A, have demonstrated that internal protein genes can dramatically influence viral fitness [15],[16],[17],[18]. Because they have segmented genomes, RVs are capable of undergoing gene reassortment during co-infection. These exchanges result in progeny virions with dsRNA segments belonging to more than one strain. However, due to the limited available sequence information, the extent to which reassortment occurs in nature is unknown. Consequently, there is an impetus to deduce the complete-genome sequences of individual RV isolates to better understand the evolutionary dynamics of this medically important pathogen.

Recent studies by our laboratory and others have made some initial strides in the area of RV genomics. In particular, Matthijnssens et al. analyzed hundreds of human and animal RV gene sequences, including 35 complete genomes, to devise a novel classification and nomenclature system [5],[6]. Their results show that most human RV isolates have genes similar in sequence to those of prototype genogroup strains Wa (genotype 1 genes) or DS-1 (genotype 2 genes) [5]. A RV is classified as a pure Wa or DS-1 genogroup member if its nine internal protein genes (those encoding VP1-3, VP6, and NSP1-5) are genotype 1 or 2, respectively. If an isolate contains both genotype 1 and 2 genes, it is considered an inter-genogroup reassortant. The G1P[8], G3P[8], G4P[8], and G9P[8] primary isolates and laboratory strains (cell-culture adapted), for which sequences are available, have only genotype 1 genes and, therefore, belong to the Wa genogroup [5]. In a similar manner, all known G2P[4] laboratory strains have only genotype 2 genes and are considered DS-1 genogroup strains [5]. The unusual human RV G/P-types (G6, G8, G9, G10, G12, P[6], P[9], and P[14]) are more often inter-genogroup or inter-species reassortants [5],[14]. It is not clear why some human RV G/P-types have ‘pure genogroup’ genome constellations, but it is possible that viral genes have co-evolved to create protein sets that operate best when kept together. In support of this idea, Heiman et al. found that genotype 1- or 2-specific amino acids cluster in definitive regions of viral proteins, many of which are sites of known protein-protein interactions [19]. It is important to note, however, that the genomes of very few primary G1P[8], G2P[4], G3P[8], G4P[8], or G9P[8] clinical isolates have been sequenced, making it difficult to ascertain whether preferred genome constellations are seen in human RVs that normally cause disease.

Indeed, the lack of complete-genome sequence data has made it hard to answer some key questions related to RV diversity and evolution: Does reassortment readily occur between genogroups in nature? Do genetically distinct clades of the same G/P-type exist and do they co-circulate during an epidemic season? Is there a correlation between genome constellation and G/P-type dominance? Towards answering these questions, we sequenced the complete genomes of G3P[8] RVs found in fifty-one archival stool samples, which were collected longitudinally (1974 to 1991) from sick children at Children's Hospital National Medical Center in Washington, DC [20],[21],[22],[23] (N. Santos et al., unpublished data). The data presented in this report, derived from the first large-scale RV genomics project, provide exceptional insight into the evolution of group A RVs and their pattern of transmission through the human population.

## Results/Discussion

### Complete-genome sequencing of fifty-one G3P[8] RVs

During the years of 1974 to 1991, stool specimens were collected from infants and young children who were hospitalized with gastroenteritis at Children's Hospital National Medical Center [20],[21],[22],[23] (N. Santos et al., unpublished data). RNA was extracted from RV-positive samples and classified into G/P-types by polymerase chain reaction (PCR)-enzyme-linked immunosorbent assay (ELISA) [24] (N. Santos et al., unpublished data). Consistent with the distribution typically seen in the United States, G1P[8], G2P[4], G3P[8], and G4P[8] strains were predominant, making up 83% of the total samples (Table 1). The incidence of G1P[8] was by far the highest, averaging 56% of the total samples, with G3P[8] strains being the second most prevalent at 20%. However, in 1976 and 1991, the frequency of G1P[8] strains was much lower than normal (11% and 26%, respectively), which correlated with increased detection of G3P[8] strains (65% and 40%, respectively) (Table 1).

**Table 1:** Distribution of RV G/P-types from Sick Children in Washington, DC (1974–1991).

Year	#Samples (% Total)				
	G1P[8]	G2P[4]	G3P[8]	G4P[8]	Other
1974	20 (71)	0 (0)	1 (4)	1 (4)	6 (21)
1975	31 (71)	1 (2)	8 (18)	0 (0)	4 (9)
1976	8 (11)	2 (3)	49 (65)	2 (3)	14 (19)
1977	20 (40)	6 (12)	4 (8)	8 (16)	12 (24)
1978	55 (79)	1 (1)	11 (16)	1 (1)	2 (3)
1979	44 (67)	8 (12)	6 (9)	1 (2)	7 (11)
1980	37 (46)	3 (4)	10 (13)	18 (23)	12 (15)
1981	5 (100)	0 (0)	0 (0)	0 (0)	0 (0)
1987	6 (46)	0 (0)	0 (0)	0 (0)	7 (54)
1988	112 (78)	0 (0)	7 (5)	1 (1)	23 (16)
1989	60 (75)	0 (0)	8 (10)	0 (0)	12 (15)
1991	30 (26)	6 (5)	46 (40)	0 (0)	33 (29)
**Total**	**428 (56)**	**27 (4)**	**150 (20)**	**32 (4)**	**132 (17)**

Due to the fluctuating pattern of occurrence, the G3P[8] strains were chosen for complete-genome sequence analysis and RNA was extracted from 150 stool samples. Of these 150 samples, 51 contained viral RNA of sufficient quantity and quality to obtain complete-genome sequences using a reverse transcription (RT)-PCR-sequencing pipeline at the J. Craig Venter Institute (JCVI), Rockville, MD. Primers were designed based on the human strain P, the only G3P[8] RV genome completely sequenced to date, and were refined iteratively as data was generated during this project [19]. Nucleotide sequences of all eleven gene segments were derived for fifty-one of the samples. The open-reading frames (ORFs) of the gene segments were determined and, for some of the genes/samples, sequences for portions of the conserved 5′ and 3′ untranslated regions were also deduced (Table 2). A few of the ORF sequences have several nucleotides missing from either of their termini; nonetheless, the coding completeness for each gene is between 99.1 and 100% (Table 2). Multiple, overlapping reads (7.6 to 10.6 times coverage) were determined, and the derived sequences showed no evidence of heterogeneity, suggesting that each stool specimen likely contained a single, dominant RV isolate (Table 2).

**Table 2:** Sequencing Results for Fifty-one Complete Genomes of Human G3P[8] RVs.

Gene	ORF size (nt)	Sequenced (nt)	ORF (% complete)	Coverage (X)
VP1	3,267	3,263	99.9	10.5
VP2	2,691	2,667	99.1	8.9
VP3	2,508	2,517	100	8.2
VP4	2,328	2,332	100	9.0
VP6	1,194	1,308	100	7.8
VP7	981	992	100	12.0
NSP1	1,461	1,496	100	8.3
NSP2	954	997	100	8.9
NSP3	933	1,009	100	10.6
NSP4	528	682	100	7.6
NSP5	594	620	100	9.6
**Per Isolate**	**17,439**	**17,888**		
**Total**	**889,389**	**912,293**		

### Multiple clades of Wa genogroup G3P[8] RVs co-circulate

G3 RV strains are quite ubiquitous in nature and have been isolated from various animal species (monkeys, dogs, cats, pigs, cattle, lambs, goats, horses, rabbits, mice and humans) [5],[25]. The complete genomes of several animal (SA11, RRV, TUCH, 30/96, CU-1, K9, A79-10, Cat2, Cat97 and A131) and human (P, AU-1, Ro1845, HRC3A, and B4106) G3 strains have been sequenced and the individual genes classified into genotypes according to the system established by the Rotavirus Classification Working Group (RCWG) [5],[6],[26]. Using this system, it was shown that G3 strains have very divergent genome constellations and contain genes belonging to several genotypes (Table S1). Specially, the human G3P[8] strain P contains exclusively genotype 1 genes, making it a pure Wa genogroup virus, whereas the human G3P[9] strain AU-1 contains all genotype 3 genes (Table S1). Strains B4106 (G3P[14]), Ro1845 (G3P[3]), and HCR3A (G3P[3]) were isolated from humans following a interspecies transmission events, and contains human genotype 2 (DS-1-like) and genotype 3 genes, as well as animal RV-like genes (Table S1).

Given the broad host range and observed genetic diversity for G3 serotype strains, we wondered whether the Washington, DC G3P[8] RVs contain genes belonging to a single or multiple genotypes. Using the RCWG classification system, we confirmed the G/P-types of the viruses and found that the other nine genes of the G3P[8] strains can be classified as genotype 1 (G3-P[8]-I1-R1-C1-M1-A1-N1-T1-E1-H1) (Table S1). Thus, none of the fifty-one G3P[8] RVs are predicted to be inter-genogroup or inter-species reassortants, but instead can be considered pure Wa genogroup strains. Preliminary analysis of G2P[4] RVs from this same collection showed they contain only genotype 2 genes, allowing them to be classified into the DS-1 genogroup (A. Rolle et al., unpublished data). This observation suggests that, although opportunities for inter-genogroup reassortment theoretically existed, there appears to have been strong biases towards the maintenance of pure Wa-like genome constellations for the G3P[8] isolates.

**Figure 1:**
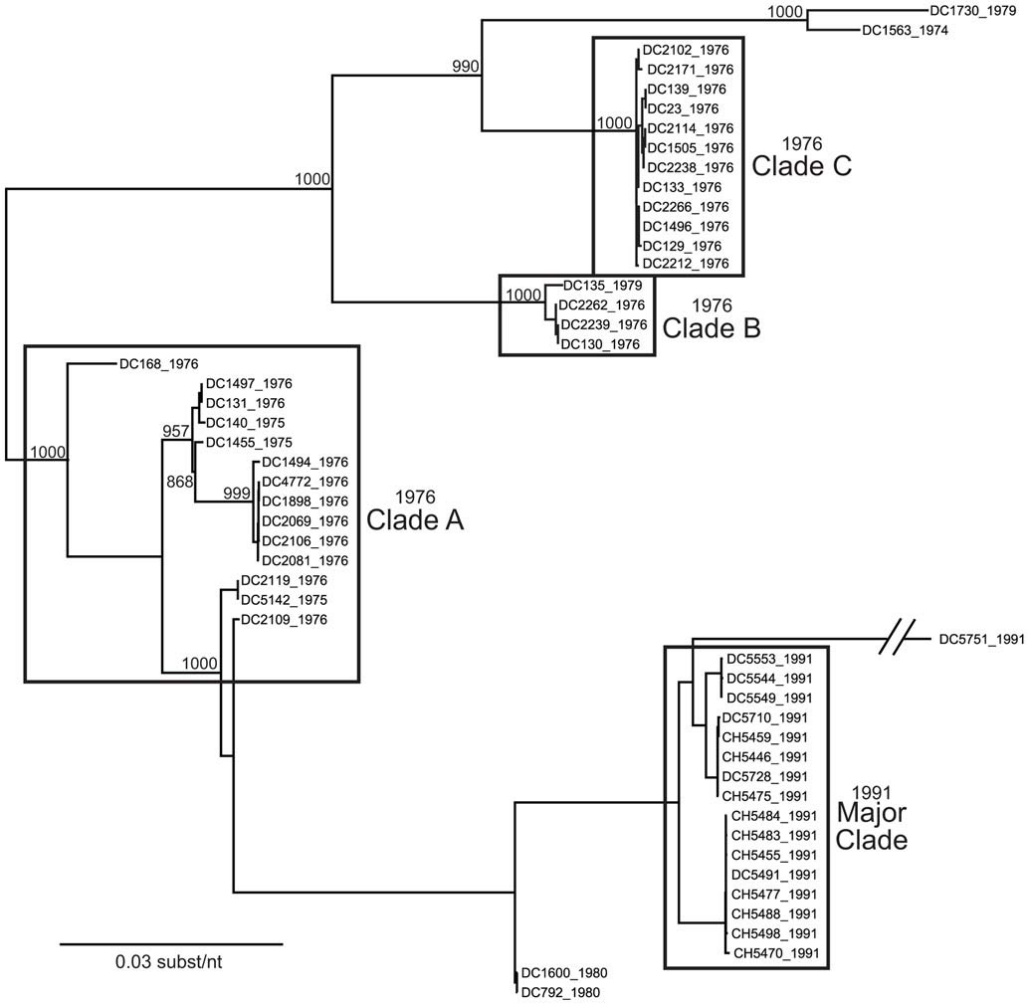
Phylogenetic Relationships Among the Genomes of the G3P[8] RVs Sampled in Washington, DC during 1976–1991. The maximum likelihood tree was constructed using the concatenated ORF nucleotide sequences for each isolate and is mid-point rooted for purposes of clarity. Horizontal branch lengths are drawn to scale, and bootstrap values are shown as percentages for key nodes. Isolates assigned into clades (1976 clades A, B, and C; 1991 major clade) are indicated by boxes.

To investigate the genetic relatedness of the fifty-one G3P[8] RVs, we reconstructed phylogenetic trees using concatenated gene sequences (i.e., genome sequences) (Figure 1). The results showed that the sequences did not necessarily cluster in a manner consistent with the year of sample collection. In particular, the genome sequences of RVs from the 1976 season appear related to those of from 1974, 1975, or 1979 strains and can be grouped into three distinct clades (A, B, and C) (Figure 1). The genome sequences of these earlier G3P[8] isolates seem more distantly related to those of the 1980 and 1991 isolates, which cluster closely together (Figure 1). An exception in 1991 is DC5751; this strain is an outlier and does not belong to the 1991 major clade (Figure 1). Due to the very limited number of samples from 1974, 1975, 1979, and 1980, it is difficult to determine the relationship among RVs within each of these years. Nonetheless, it is clear that multiple clades of Wa genogroup G3P[8] RVs co-circulated and caused disease in the 1976 season, but that the 1991 season was dominated by a single G3P[8] clade.

### Intra-genogroup reassortment versus preferred genome constellations

**Figure 2:**
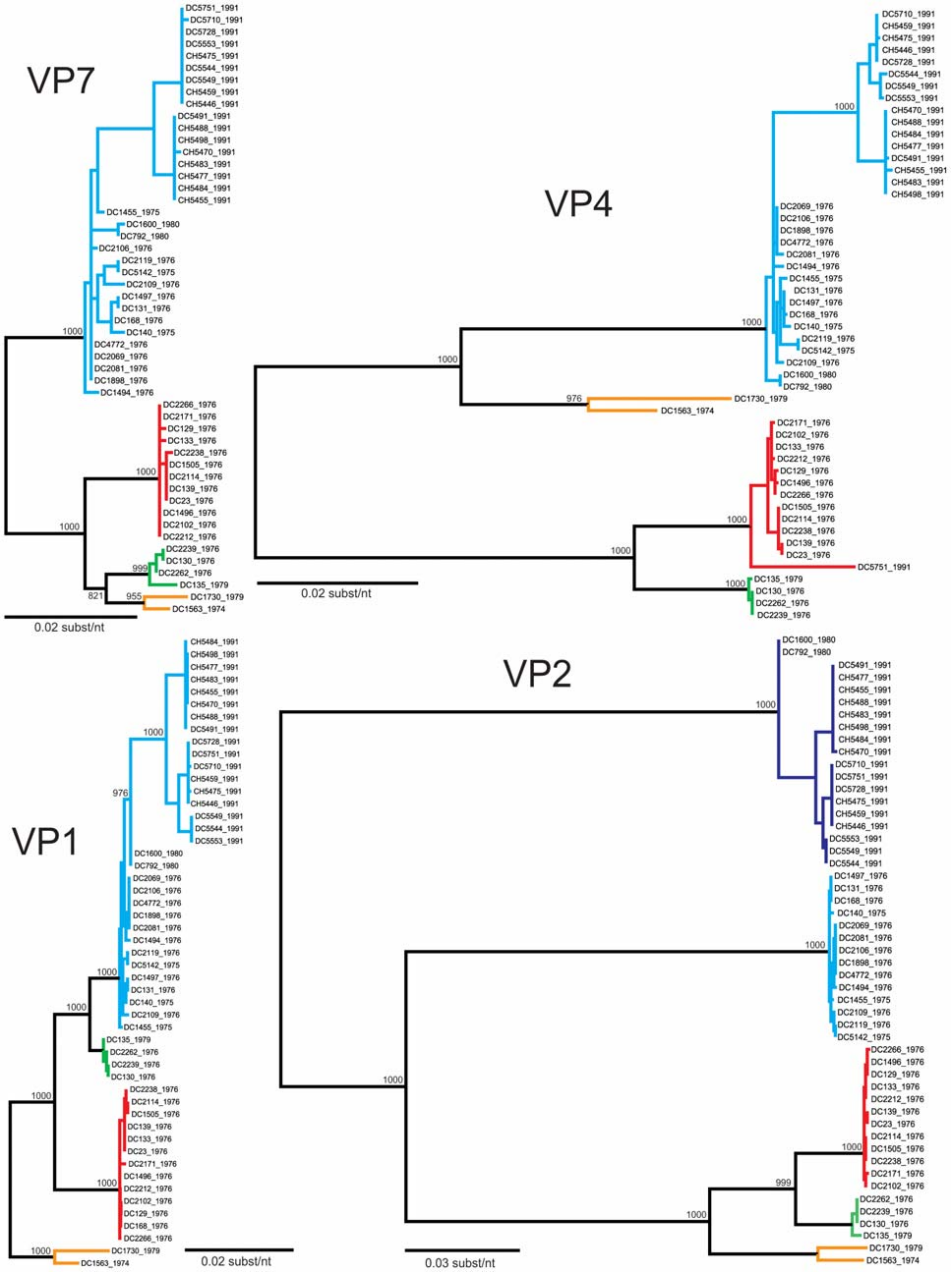
Phylogenetic Relationships Among the Individual Genes of the G3P[8] RVs: VP7, VP4, VP1, and VP2. The maximum likelihood trees were constructed using the ORF nucleotide sequences for each gene of each isolate and are mid-point rooted for purposes of clarity. All horizontal branch lengths are drawn to scale, and bootstrap values are shown per 1000 replicates for key nodes. Sequences that cluster together in all algorithms tested were assigned the same color (orange, green, red, cyan, or navy blue), and separate colors indicated divergent gene alleles.

The genetic variability among the G3P[8] strains found in the genome tree can either be attributed to the accumulation of point mutations over time (genetic drift) or to gene reassortment within the Wa genogroup (genetic shift). To determine the contribution of these two evolutionary mechanisms, we performed phylogenetic analyses for each gene (Figures 2–4). The results using a maximum likelihood analyses are presented in Figures 2–4; however, the overall tree topologies were nearly identical when neighbor-joining analyses or bayesian inference were used (data not shown). Clusters of sequences in the individual gene trees represent genetically-distinct alleles and have been color-coded based on the consensus of all phylogenetic algorithms tested. Reassortment events are indicated by (i) the movement of a particular isolate from one color-grouping to another or (ii) the emergence of new color-groupings.

**Figure 3 ppat-1000634-g003:**
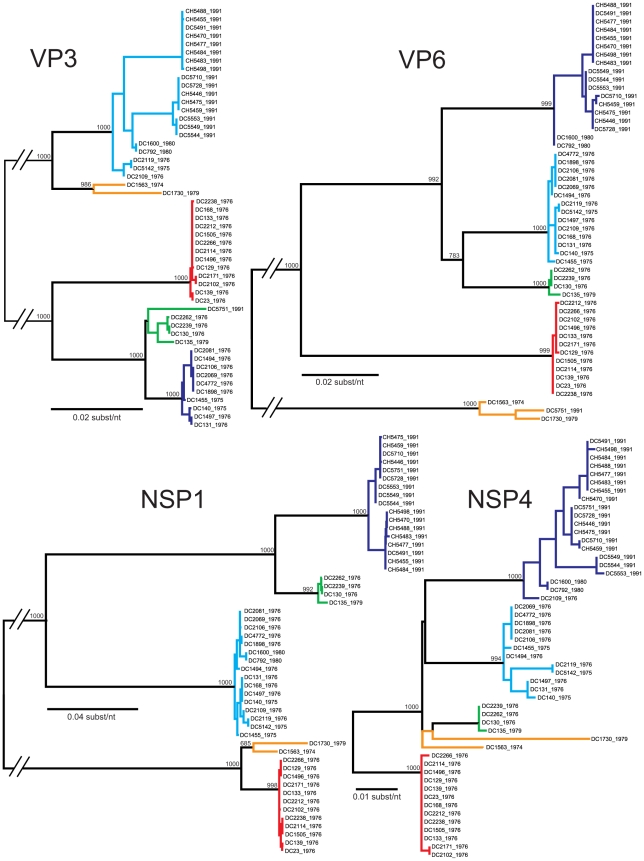
Phylogenetic Relationships Among the Individual Genes of the G3P[8] RVs: VP3, VP6, NSP1, and NSP4. Details are given in the legend for Figure 2. Horizontal branch lengths are drawn to scale, expect for those interrupted with hash bars, which have been truncated.

**Figure 4:**
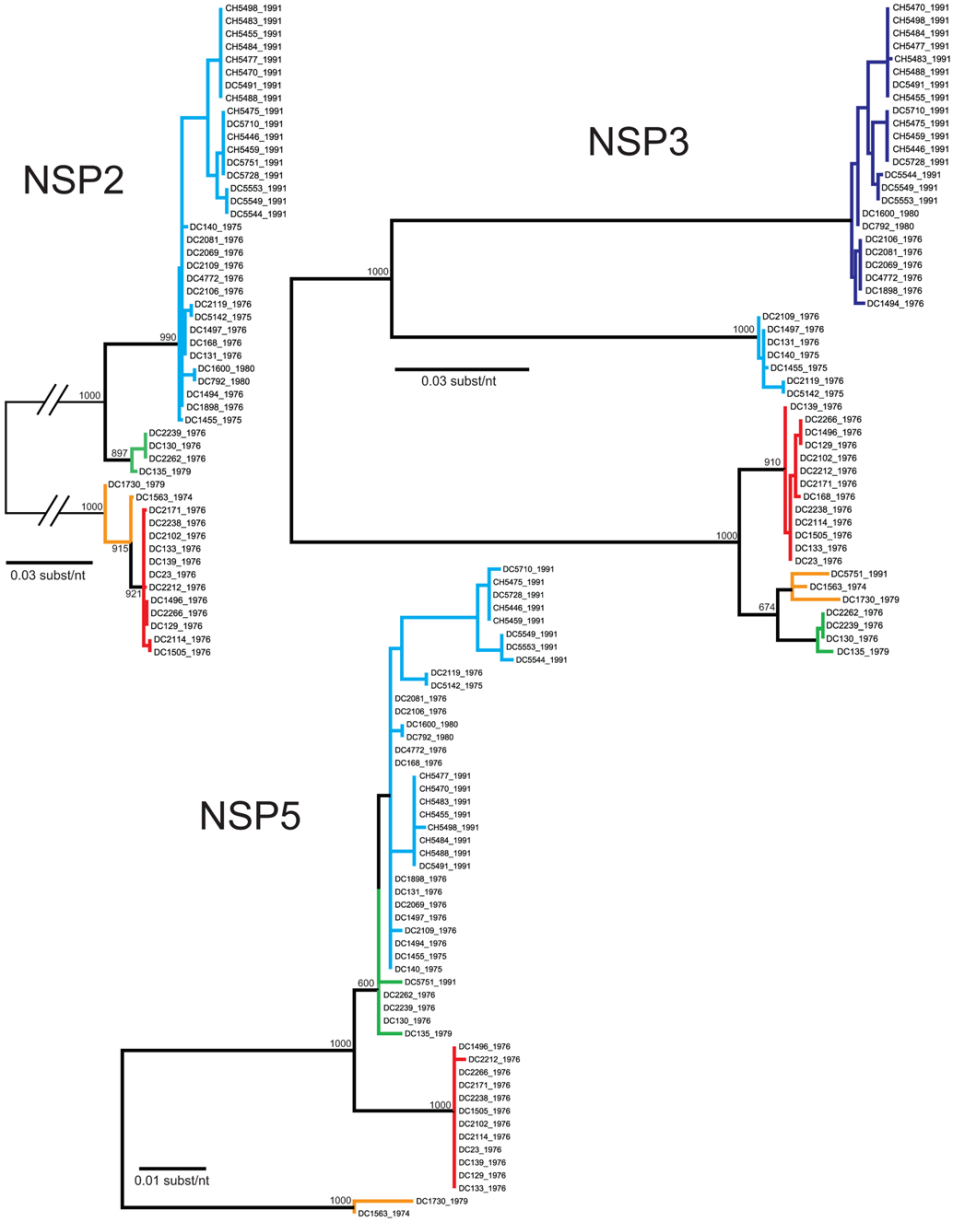
Phylogenetic Relationships Among the Individual Genes of the G3P[8] RVs: NSP2, NSP3, and NSP5. Details are given in the legends for Figures 2 and 3.

**Figure 5:**
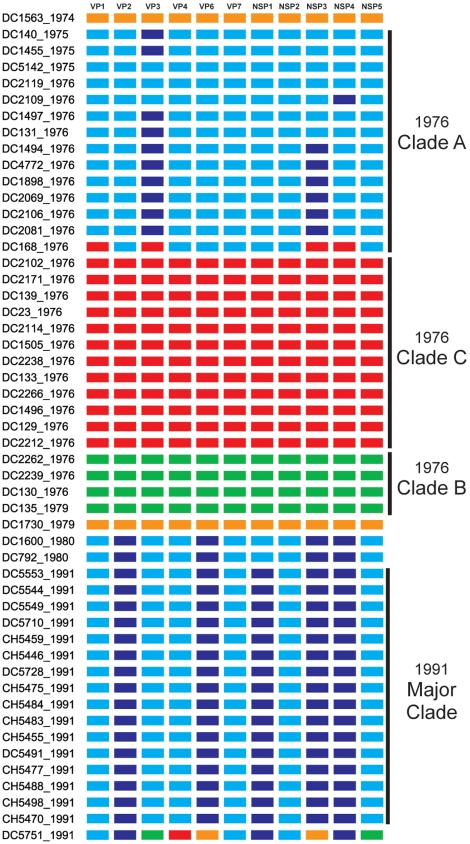
Allele-based Genome Constellations of the G3P[8] RVs. The schematic illustrates the color-coding of each gene allele for the G3P[8] isolates based on the phylogenies shown in Figures 2–4. The sample name and year of isolation is listed to the left of the corresponding genome constellation. The protein encoded by each gene is listed at the top. Isolates assigned into clades (1976 clades A, B, and C; 1991 major clade) are indicated.

The summary of the individual gene phylogenies is consistent with the results using the genomes and reveals the true genetic make-up of the G3P[8] isolates (Figure 5). Each gene can be classified into four or five alleles (orange, green, red, cyan, or navy blue) that are defined by strong bootstrap (neighbor-joining or maximum likelihood analyses) or posterior probabilities (bayesian inference) (Figures 2–4 and data not shown). Twenty of the fifty-one isolates (39%) show genes that fall into the same topological position on each tree and maintain the same color, indicating the lack of intra-genogroup reassortment. For example, the 1976 clades B and C have pure-color genome constellations (green and red, respectively), demonstrating that they each evolved distinctly and did not recently share genetic information (Figure 5). Likewise the 1974 and 1979 isolates (DC1563 and DC1730; orange), as well as the 1975 and 1976 isolates (DC5142 and DC2119; cyan) are not predicted to be reassortants (Figure 5). This observation was surprising and suggests that, even within the Wa genogroup not all gene allele combinations are tolerated and there may be pressure(s) to maintain certain genome constellations. Nonetheless, thirty-one of the isolates (61%) can be described as intra-genogroup reassortants, containing gene sequences with different phylogenetic patterns and, therefore, belonging to more than one color group (Figure 5). The majority of these reassortants belong to the 1976 clade A or the 1991 major clade and exhibit a cyan background with several navy blue alleles (Figure 5). None of the isolates from our collection show a predominant navy blue genome constellation, suggesting that these gene alleles may have been picked up from a G3P[8] strain not sequenced in this study or from co-circulating non-G3P[8] Wa genogroup viruses. The two other types of reassortants detected include: (i) the 1976 isolate (DC168; clade A), which contains gene alleles belonging to the cyan and red color groups and (ii) the 1991 isolate (DC5751), which contains gene alleles belonging to all five color groups (orange, green, red, cyan, or navy blue) (Figure 5). The 1991 DC5751 isolate is particularly interesting, as several of its gene alleles (orange, green, and red) are not detected in the co-circulating G3P[8] RV population. We predict that, similar to the navy blue alleles seen in 1975, 1976, 1980, and 1991 strains, the orange, green, and red alleles of DC5751 were donated by a Wa genogroup strain belonging to a different G/P-type. Future studies aimed at elucidating the genome constellations of the G1P[8] and G4P[8] strains from this archival stool collection will shed light on (i) whether these isolates also belong to the Wa genogroup and (ii) whether they exchanged genes segments with the G3P[8] population. Still, the results presented in this report suggest that, although genetic shift plays an important role in RV evolution, reassortment among viral genes that have become divergent due to genetic drift may create less fit genome constellations.

### Identification of allele-specific amino acid differences

Having observed that G3P[8] RVs encode genes that are genetically-divergent at the nucleotide level, we next sought to determine whether the color-coded alleles also encode different proteins. In particular, to identify the precise residues that distinguish proteins from the orange, green, red, cyan, or navy blue alleles, amino acid alignments were constructed. Residues in the alignments that differ depending on the color grouping of the gene (i.e., allele-specific differences) were identified (Table S2). We found that, indeed, the grouping based on nucleotide analyses correlate significantly with changes in amino acid sequence. Proteins VP1, VP6, VP7, NSP2, NSP4, and NSP5 are highly conserved among the G3P[8] RVs, showing only 6 to 15 allele-specific amino acid differences (Table S2). In contrast, proteins VP2, VP3, VP4, and NSP3 are more variable and exhibit 27 to 41 differences (Table S2). By far the most variation was seen in NSP1, which functions as an innate immune antagonist [27],[28]. We found 101 allele-specific differences for this non-structural protein; the basis of such extreme variation is not known. The majority of the amino acid changes among the G3P[8] RVs of this study are also found in other published RV sequences. However, a few of the gene alleles show unique residues that have not been seen in any human or animal RV strain sequenced to date (Table S2; yellow-shading).

**Figure 6:**
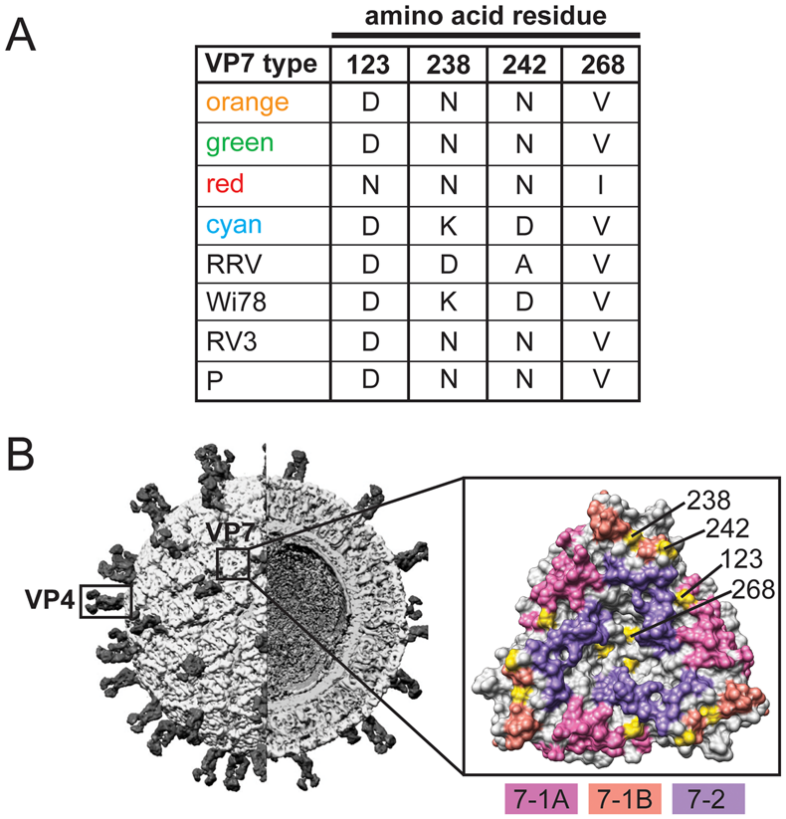
Surface-Exposed Allele-Specific Amino Acid Differences for VP7. (A) Table of allele-specific differences located on the surface of RRV VP7. The VP7 type corresponds to the alleles defined in Figure 2 (orange, green, red, and cyan) or to reference G3 RV strains (RRV, Wi68, RV3 and P). The amino acid at each position is listed to the right of the VP7 type. Numbering is based on RRV VP7 (GenBank# AF295303). (B) Three-dimensional location of surface-exposed allele-specific differences. The left image shows the architecture of a RV virion (modified with permission from B.V.V. Prasad) and the positions of VP7 and VP4. The right image shows a surface representation of the VP7 trimer crystal structure (PDB# 3FMG). Residues comprising the putative neutralization domains of VP7 are listed in Table S3 and have been colored as follows: pink (7-1A), salmon (7-1B), and purple (7-2). Allele-specific differences are shown in yellow and are labeled for a single monomer of the trimer.

The high-resolution structures of G- and P-type antigens (VP7 and VP4, respectively) of rhesus RV (strain RRV) have been solved, affording the opportunity to map the three-dimensional locations of the allele-specific differences [29],[30],[31],[32]. For the VP7 glycoprotein, three putative neutralization domains (7-1A, 7-1B, and 7-2) are predicted based on amino acid alignments and the mapping of monoclonal antibody escape mutants (Table S3) [29]. Of the six allele-specific differences in VP7, four of them are located on the surface of the protein, in or near neutralization domains (Figure 6). In particular, domain 7-1A shows a single allele-specific difference (RRV residue 123), while two differences are located within domain 7-1B (RRV residues 238 and 242). A single amino acid change (RRV residue 268) is also seen proximal to neutralization domain 7-2. Although these differences are not sufficient to change the G-type of these viruses, they may subtly affect binding of neutralizing antibodies and confer a selective pressure that influences viral fitness. Additionally, the G3 component of the pentavalent RV vaccine RotaTeq (strain Wi78) or those of vaccines currently being developed (strains RV3 and P) seem to match some VP7 proteins better than others (Figure 6 and Table S2) [33],[34],[35],[36],[37]. Because the allele-specific differences identified in this study are also seen in VP7 sequences of present-day G3 RV strains, it will be important to learn whether they are determinants of vaccine efficacy.

**Figure 7:**
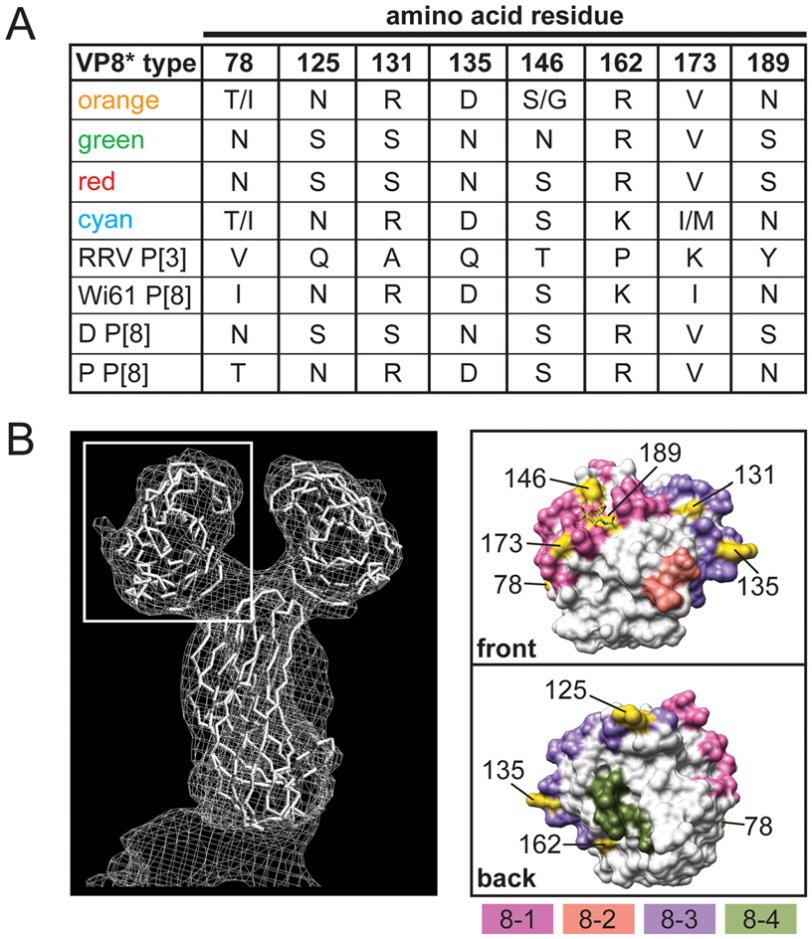
Surface-Exposed Allele-Specific Amino Acid Differences for VP8*. (A) Table of allele-specific differences located on the surface of RRV VP8*. The VP8* type corresponds to the alleles defined in Figure 2 for VP4 (orange, green, red, and cyan), to the P[3] VP4 RRV, or to the P[8] RV strains Wi61, D and P. The amino acid at each position is listed to the right of the VP8* type. Numbering is based on RRV VP4 (GenBank# AY033150). (B) Three-dimensional location of surface-exposed allele-specific differences. The left image shows a surface representation of the VP4 crystal structure (PDB# 1KQR). A white box defines the position of VP8*. The right images show the VP8* core from two different viewpoints (front or back). The front viewpoint is rotated 90° to the right compared with the image in the white box. The back viewpoint is rotated 180° to the left compared with the front. Residues comprising the putative neutralization domains of VP8* are listed in Table S4 and have been colored as follows: pink (8-1), salmon (8-2), purple (8-3), and olive (8-4). Allele-specific differences are shown in yellow.

**Figure 8:**
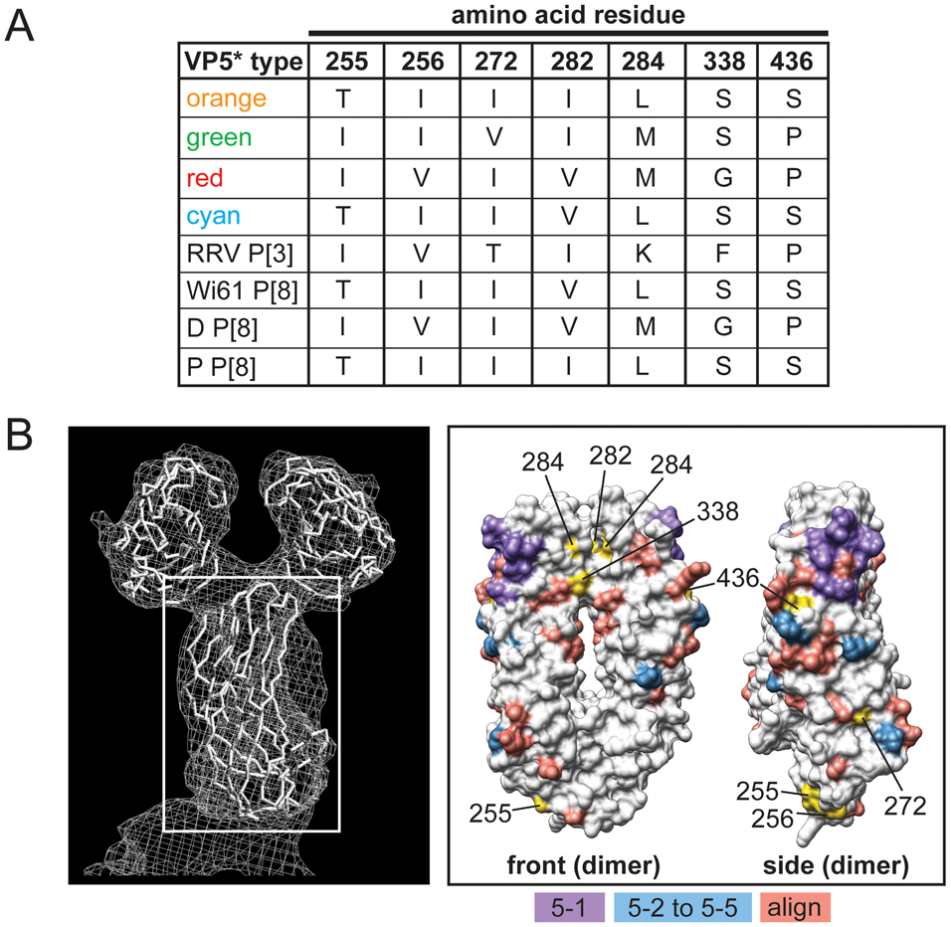
Surface-Exposed, Allele-Specific Amino Acid Differences for VP5*. (A) Table of allele-specific differences located on the surface of RRV VP5*. The VP5* type corresponds to the alleles defined in Figure 2 for VP4 (orange, green, red, and cyan), to the P[3] VP7 RRV, or to the P[8] RV strains Wi61, D and P. The amino acid at each position is listed to the right of the VP5* type. Numbering is based on RRV VP4 (GenBank# AY033150). (B) Three-dimensional location of surface-exposed allele-specific differences. The right image shows a surface representation of the VP4 crystal structure (PDB# 1KQR). A white box defines the position of VP5*. The left images show the VP5* dimer from two different viewpoints (front or side) (PDB# 2B4H). The front viewpoint is rotated 90° to the left compared with the image in the white box. The side viewpoint is rotated 90° to the right compared with the front. Residues comprising the putative neutralization domains of VP5* are listed in Table S5 and have been colored as follows: purple (5-1), blue (5-2, 5-3, 5-4, and 5-5), and salmon (predicted based on alignments; align). Allele-specific differences are shown in yellow.

The VP4 spike protein is comprised of two structurally-distinct regions (VP8* and VP5*) generated following trypsin activation of the virion particle [3]. The VP8* region contains four putative neutralization domains (8-1, 8-2, 8-3, and 8-4) defined by amino acid alignments and mapping of monoclonal antibody escape mutants [30],[31],[32] (Table S4). We found that of the 13 allele-specific differences in VP8*, four of them map to domain 8-1 (RRV residues 78, 146, 173, and 189) and four of them to domain 8-3 (RRV residues 125, 131, 135, and 162) (Figure 7). Changes at position 189 may be particularly important, as they are predicted to influence sialic acid receptor binding [31]. Compared with VP8*, the regions of VP5* involved in neutralizing antibody binding have not been well characterized, but the mapping of escape mutants have identified several important domains (Table S5). Additionally, amino acid alignments highlighted P[8]-specific residues of VP5* that might play a role in virus neutralization. We found ten allele-specific amino acid changes in VP5*, seven of which (RRV residues 255, 256, 272, 282, 284, 338, and 436) are surface-exposed in either the native or trypsin-activation form of the protein. Based on its three-dimensional location, residue 436 is most likely to influence neutralizing antibody binding (Figure 8). Similar to the changes seen in VP7, those in VP4 are not predicted to alter the P-type classification of these strains, and they represent the diversity seen modern day P[8] RVs. The sequences of the VP4 P[8] components of the RV vaccines RotaTeq (strain Wi79) and Rotarix (strain 89-12) are not available to the public [33],[38]. Therefore, we cannot predict if or how these allele-specific changes might affect vaccine efficacy.

### Summary and conclusions

RVs continue to be a primary cause of childhood diarrheal illness and are associated with significant morbidity and mortality, particularly in developing countries. Despite their medical importance, the lack of sequence information has hindered our understanding of how RVs evolve during and between epidemic seasons. Genome sequences of several laboratory strains have allowed for the development of classification systems based on the outer capsid protein genes (G/P-types) or the internal protein genes (genogroups) [5],[6]. RVs with certain G/P-types (such as G1P[8], G3P[8], and G4P[8]) tend to contain all genotype 1 internal protein genes and belong to the Wa genogroup. In contrast, strains classified as G2P[4] seem to have only genotype 2 genes and to belong to the DS-1 genogroup. Although inter-genogroup reassortants exist, emerging evidence suggests that such mixed genome constellations (those containing both genotype 1 and 2 genes) may be less fit and selected against in nature. In support of this notion, we found that the genomes of fifty-one G3P[8] primary RV isolates are comprised only of genotype 1 genes, despite the fact that viruses containing genotype 2 genes (i.e., G2P[4] strains) were present during the same epidemic season (A. Rolle et al., unpublished). It is possible that G2P[4] and G3P[8] strains did not physically co-infect children, thereby preventing the opportunity for reassortment. However, we think it is more likely that both co-infection and inter-genogroup reassortment occurred, but that the resultant viruses were unable to compete with parental strains. Thus, the natural selection of isolates containing genes that operate best when kept together may limit the amount of observed genetic shift that occurs during RV evolution. In essence, RVs must balance the advantages of gene reassortment with the disadvantages of unlinking preferred genes/protein combinations. This observation is in contrast to what has been generally reported for influenza A viruses, for which ongoing, robust reassortment is evident with limited evidence of genetic linkages among gene segments for those viruses infecting a common animal species [18],[39],[40],[41].

In addition to reassortment biases between the DS-1 and Wa genogroups, our results also suggest that there may be preferences towards the maintenance of certain genome constellations even for RVs belonging to the same genogroup. Although the genes of the fifty-one G3P[8] RVs are technically genotype 1, we found significant variation among them and were able to classify each into four or five distinct alleles. Many of the isolates show pure color genome constellations, indicating the lack of intra-genogroup reassortment. Of the isolates that do show evidence of genetic exchange, most did not reassort with co-circulating G3P[8] strains. Instead, we predict that the majority of reassortants pick up genes (such as the navy blue alleles) from other Wa genogroup viruses belonging to different G/P-types (G1P[8] or G4P[8]). The lack of robust, ongoing reassortment may have resulted in the maintenance of the 1976 G3P[8] RVs as genetically distinct, stable, co-circulating clades (A, B, and C). Importantly, the 1976 clades have amino acid differences in all eleven viral proteins. It is possible that allele-specific residues contributed to the evolution of genome constellations encoding more fit protein sets, much like what is seen at the genogroup level. In this manner, only intra-genogroup reassortants with gene alleles encoding compatible proteins (such as cyan and navy blue) emerged in the G3P[8] RV population. Moreover, by mapping the allele-specific amino acid differences onto the high-resolution structures of serotype antigens VP7 and VP4, we found that several are located in or near putative neutralization domains. By having outer capsid proteins that are slightly different, and possibly more capable of mediating cell entry or evading the host immune response, the 1976 clade A viruses may have had a selective advantage. This advantage would explain why the 1991 epidemic season was characterized by a single clade of viruses whose outer capsid proteins are remarkably similar to those of the 1976 clade A strains. The observed diversity in VP7 and VP4 of the fifty-one archival G3P[8] RVs sequenced in this study mirrors what is seen in currently circulating strains. The results presented in the current report are expected to provide a foundation for future studies aimed at elucidating the role, if any, these amino acid changes have on viral fitness and vaccine efficacy.

## Materials and Methods

### Sample collection, RNA extraction, and G/P-typing

The study population included infants and young children who were hospitalized with diarrhea at Children's Hospital National Medical Center, Washington, DC [20],[21],[23]. Fecal specimens (rectal swabs or diaper scrapings) were collected and tested for evidence of RV using electron microscopy and for viral antigen using ELISA. RNA was extracted from RV-positive samples using TRIzol (Invitrogen) and samples were classified into G/P-types based on the results of a microtiter plate hybridization-based PCR-ELISA [24] (Santos et al, unpublished data). The isolated RNA was subsequently used for RT-PCR and nucleotide sequencing as described below.

### RT-PCR and nucleotide sequencing

Oligonucleotide primers were initially designed based on the human RV strain P [19] and then improved iteratively as new sequence data was generated. Primers were designed every 600 bp along both sense and antisense strands to provide greater than 4 times (4×) coverage by RT-PCR. An M13 tag was added to the 5′ end of each primer (sense: TGTAAAACGACGGCCAGT; antisense CAGGAAACAGCTATGACC) for use in sequencing (see below).

RT-PCRs were performed with 1 ng of RNA using OneStep RT-PCR kits (Qiagen) according to manufacturer's instructions with minor modifications. Reactions were scaled down to 1/5 the recommended volumes, the RNA templates were denatured in 50% DMSO at 95°C for 5 min, and 1.6 units RNase Out (Invitrogen) was added. Following RT-PCR cycling, the reactions were treated with 0.5 units of shrimp alkaline phosphatase and 1 unit of exonuclease I (USB) incubation at 37°C for 60 min to inactivate remaining deoxyribonucleotides and digest the single-stranded primers. Enzymes were heat inactivated by incubation at 72°C for 15 min.

The RT-PCR products were sequenced with an ABI Prism BigDye v3.1 terminator cycle sequencing kit (Applied Biosystems) using M13 primers (listed above). The dye terminator was removed using Performa DTR (Edge Biosystems) and sequences were obtained with a 3730 DNA Analyzer (Applied Biosystems). Raw sequence data was trimmed to remove any primer-derived sequence as well as low quality sequence, and gene sequences were assembled using the Elvira and TIGR assemblers (www.jcvi.org/cms/research/software). The gene sequences were then manually edited using CloE (Closure Editor; JCVI) and any polymorphisms were re-analyzed by sequencing. Finally, the Viral Genome ORF Reader (VIGOR; JCVI) program was used to: check segment length, perform alignments, ensure the fidelity of open-reading frames, correlate nucleotide polymorphisms with amino acid changes, and detect any potential sequence errors.

### Phylogenetic and structural analyses

Maximum likelihood phylogenetic trees were reconstructed using PhyML [42] employing the Hasegawa-Kishino-Yano substitution model (HKY85) and gamma-distributed rate variation among sites. Bootstrap analysis was performed based on 1000 replicates and trees were visualized using FigTree (http://tree.bio.ed.ac.uk/software). Amino acid alignments were constructed with MacVector 8.1.2. (Accelrys) using ClustalW, BLOSUM Series, with the defaults set (open gap penalty of 10.0, extended gap penalty of 0.05, and delay divergence of 40%). The sequence of strain Wi78 VP7 was found in Nishikawa et al. [37]. Structural analysis of VP7 (PBD# 3FMG), VP8* (PDB# 1KQR), and VP5* (PDB# 2B4H), was performed using UCSF Chimera-Molecular Molecular Modeling System [43].

Accession numbers of published protein sequences used in this study include: strain RRV VP7 and VP4 (AF295303 and AY033150, respectively); strain RV3 VP7 (FJ998278); strain D VP4 (EF672570); strain P (EF583037–EF583037 and EF67598–EF67604); strain Wi61 (EF583049–EF583052 and EF672619–EF672625); strain IAL28 (EF583029–EF583032 and EF672584–EF672590), strain DS-1 (EF583025–EF583028 and EF672577–EF672583).

Accession numbers deposited into GenBank include: DC1563_1974 (FJ947175–FJ947185); DC140_1975 (FJ947738–FJ947748); DC1455_1975 (FJ947186–FJ947196); DC5142_1975 (FJ947197–FJ947207); DC2119_1976 (FJ947395–FJ947405); DC2109_1976 (FJ947373–FJ947383); DC1497_1976 (FJ947340–FJ947350); DC131_1976 (FJ947252–FJ947262); DC1494_1976 (FJ947274–FJ947284); DC4772_1976 (FJ947362–FJ947372); DC1898_1976 (FJ947296–FJ947306); DC2069_1976 (FJ947804–FJ947814); DC2106_1976 (FJ947837–FJ947847); DC2081_1976 (FJ947815–FJ947825); DC168_1976 (FJ947749–FJ947759); DC2102_1976 (FJ947826–FJ947836); DC2171_1976 (FJ947406–FJ947416); DC139_1976 (FJ947219–FJ947229); DC23_1976 (FJ947208–FJ947218); DC2114_1976 (FJ947384–FJ947394); DC1505_1976 (FJ947351–FJ947361); DC2238_1976 (FJ947417–FJ947427); DC133_1976 (FJ947263–FJ947273); DC2266_1976 (FJ947881–FJ947891); DC1496_1976 (FJ947285–FJ947295); DC129_1976 (FJ947230–FJ947240); DC2212_1976(FJ947848–FJ947858); DC2262_1976 (FJ947870–FJ947880); DC2239_1976 (FJ947859–FJ947869); DC130_1976 (FJ947241–FJ947251); DC135_1979 (FJ947307–FJ947317); DC1730_1979 (FJ947318–FJ947328); DC1600_1980 (FJ947771–FJ947781); DC792_1980 (FJ947760–FJ947770); DC5553_1991 (FJ947936–FJ947946); DC5544_1991 (FJ947505–FJ947515); DC5549_1991 (FJ947516–FJ947526); DC5710_1991 (FJ947782–FJ947792); CH5459_1991 (FJ947439–FJ947449); CH5446_1991 (FJ947428–FJ947438); DC5728-1991 (FJ947329–FJ947339); CH5475_1991 (FJ947450–FJ947460); CH5484_1991 (FJ947472–FJ947482); CH5483_1991 (FJ947914–FJ947924); CH5455_1991 (FJ947892–FJ947902); DC5491_1991 (FJ947494–FJ947504); CH5477_1991 (FJ947461–FJ947471); CH5488_1991 (FJ947483–FJ947493); CH5498_1991 (FJ947925–FJ947935); CH5470_1991 (FJ947903–FJ947913); and DC5751_1991 (FJ947793–FJ947803).

### Ethics statement

Fecal specimens were collected during 1974–1991 from infants and young children who were hospitalized with diarrhea at Children's Hospital National Medical Center, Washington, DC. Samples were de-identified and analyzed anonymously. Under these conditions, the Office of Human Subjects Research (OHSR) of the National Institutes of Health has determined that Federal regulations for the protection of human subjects do not apply to the research activities described in this study (Exemption # 3937).

## Supporting Information

Table S1 Genome Constellations of G3 RVs Defined by the RCWG
(0.05 MB PDF)

Table S2 Allele-Specific Amino Acid Differences of Proteins Encoded by the G3P[8] RVs
(0.08 MB PDF)

Table S3 Residues Defining Neutralization Domains of RRV VP7
(0.04 MB PDF)

Table S4 Residues Defining Neutralization Domains of RRV VP8*
(0.05 MB PDF)

Table S5 Residues Defining Neutralization Domains of RRV VP5*
(0.04 MB PDF)

Table S6 Primers Used for RT-PCR and Sequencing
(0.07 MB PDF)
