# Supplementary material for: Evolutionary Dynamics of Human Rotaviruses: Balancing Reassortment with Preferred Genome Constellations
Source: PLoS Pathog. 2009 Oct 23;5(10):e1000634. doi: 10.1371/journal.ppat.1000634 (PMC2760143; doi:10.1371/journal.ppat.1000634)
Supplement: Table S1 — Genome Constellations of G3 RVs Defined by the RCWG (0.05 MB PDF) [file ppat.1000634.s001.pdf]

Table S1. Sequence-based Genome Constellations of G3 RVs

Strain*	VP7	VP4	VP6	VP1	VP2	VP3	NSP1	NSP2	NSP3	NSP4	NSP5
**SA11 (si)**	G3	P[1]	I2	R2	C5	M5	A5	N5	T5	E2	H5
**RRV (si)**	G3	P[3]	I2	R2	C3	M3	A9	N2	T3	E3	H6
**TUCH (si)**	G3	P[24]	I9	R3	C3	M3	A9	N1	T3	E3	H6
**A131 (po)**	G3	P[7]	I5	R1	C2	M1	A1	N1	T1	E1	H1
**30/96 (la)**	G3	P[14]	I2	R2	C2	M3	A9	N2	T6	E5	H3
**CU-1 (ca)**	G3	P[3]	I3	R3	C2	M3	A9	N2	T3	E3	H6
**K9 (ca)**	G3	P[3]	I3	R3	C2	M3	A9	N2	T3	E3	H6
**A79-10 (ca)**	G3	P[3]	I3	R3	C2	M3	A9	N2	T3	E3	H6
**Cat2 (fe)**	G3	P[9]	I3	R3	C2	M3	A3	N1	T6	E3	H3
**Cat97 (fe)**	G3	P[3]	I3	R3	C2	M3	A9	N2	T3	E3	H6
**P (hu)**	G3	P[8]	I1	R1	C1	M1	A1	N1	T1	E1	H1
**AU-1 (hu)**	G3	P[9]	I3	R3	C3	M3	A3	N3	T3	E3	H3
**B4106 (hu)**	G3	P[14]	I2	R2	C2	M3	A9	N2	T6	E5	H3
**Ro1845 (hu)**	G3	P[3]	I3	R3	C2	M3	A9	N2	T3	E3	H6
**HCR3A (hu)**	G3	P[3]	I3	R3	C2	M3	A9	N2	T3	E3	H6
**DC RVs (hu)**	G3	P[8]	I1	R1	C1	M1	A1	N1	T1	E1	H1

*Strain name followed in parentheses by species of isolation: simian (si), porcine (po), lapine (la), canine (ca), feline (fe) or human (hu); DC RVs (hu) are those G3P[8] viruses sequenced in the current study
