# Supplementary material for: Evolutionary Dynamics of Human Rotaviruses: Balancing Reassortment with Preferred Genome Constellations
Source: PLoS Pathog. 2009 Oct 23;5(10):e1000634. doi: 10.1371/journal.ppat.1000634 (PMC2760143; doi:10.1371/journal.ppat.1000634)
Supplement: Table S2 — Allele-Specific Amino Acid Differences of Proteins Encoded by the G3P[8] RVs (0.08 MB PDF) [file ppat.1000634.s002.pdf]

Table S2. Allele-Specific Amino Acid Differences of Individual Proteins

protein	aa #	orange	green	cyan	red	RRV	Wi78	RV3	P	
VP7[a]	116	V	I	I	I	V	I	I	V	
	123	D	D	D	N	D	D	D	D	
	238	N	N	K	N	D	K	N	N	
	242	N	N	D	N	A	D	N	N	
	268	V	V	V	I	V	V	V	V	
	278	M	A	A	A	A	A	A	M	
**protein**	**aa #**	**orange**	**green**	**cyan**	**red**	**RRV**	**Wi61**	**P**	**D**	
VP4[b]	35	V	I	V	I	I	V	V	I	
	78	T/I	N	T/I	N	V	I	T	N	
	109	V	I	V	I	V	V	V	I	
	121	V	I	V	I	L	V	V	I	
	125	N	S	N	S	Q	N	N	S	
	131	R	S	R	S	A	R	R	S	
	135	D	N	D	N	Q	D	D	N	
	146	S/G	N	S	S	T	S	S	S	
	162	R	R	K	R	P	K	R	R	
	173	V	V	I/M	V	K	I	V	V	
	189	N	S	N	S	Y	N	N	S	
	199	T	T	I	T	M	I	T	T	
	236	S	P	S	P	L	S	S	P	
	255	T	I	T	I	I	T	T	I	
	256	I	I	I	V	V	I	I	V	
	272	I	V	I	I	T	I	I	I	
	282	I	I	V	V	I	V	I	V	
	284	L	M	L	M	K	L	L	M	
	294	I	V	I/M	I	E	I	I	I	
	296	F/Y	Y	F	Y	S	F	F	Y	
	338	S	S	S	G	F	S	S	G	
	339	V	I	I	I	V	V	V	I	
	436	S	P	S	P	P	S	S	P	
	515	S	A	S	A	M	S	S	A	
	561	V	I	I	I	S	I	V	I	
	578	S	N	S	N	R	S	S	N	
	581	V	I	I	I	S	I	V	I	
	585	I	L	I	L	V	I	I	L	
	588	A	A	T	A	S	T	A	A	
	593	W	W	L	W	A	L	W	W	
	622	K	K	R	K	L	R	K	K	
	690	F	F	V	F	R	V	F	F	
	696	D	N	D	N	V	D	D	N	
	698	L	L	L	F	T	L	L	F	
	699	N	D	N	D	F	N	N	D	
	709	T	A	A	A	F	A	T	A	
	714	N	D	N	D	T	N	N	D	
	739	T	T	I	T	R	I	T	T	
	751	V	M	V	M	R	V	V	M	
**protein**	**aa #**	**orange**	**green**	**cyan**	**red**	**P**	**Wi61**	**IAL28**	**DS-1**	
VP1[c]	43	A	V	A	A	A	A	A	S	
	67	D	N	D	D	D	D	D	E	
	104	A	A	A	V	A	A	V	L	

	107	L	I	I	L	L	I	L	L	
	291	M	M	M	I	M	M	M	M	
	440	P	S	P	P	P	P	P	P	
	779	D	N	N	D	D	N	D	D	
	813	N	N	D	N	N	N	N	S	
	821	V	I	I	V	V	I	V	F	
	865	V	V	V	M	V	V	V	V	
	894	S	P	P	S	S	P	S	P	
	941	K	R	K	K	K	K	K	K	
	973	G	G	S	G	G	S	G	D	
**protein**	**aa #**	**orange**	**green**	**cyan**	**navy**	**red**	**P**	**Wi61**	**IAL28**	**DS-1[e]**
**VP2[d]**	61	I	I	V	I	I	I	I	V	I
	73	D	G	D	D	G	D	D	D	D
	75	V	I	V	V	I	V	V	I	V
	95	D	D	D	N	D	D	D	D	E
	100	V	I	V	I	I	V	V	V	I
	126	R	R	R	K	R	R	R	R	K
	134	M	M	T	T	M	M	M	T	T
	135	K	K	R	K	K	K	K	K	K
	143	R/K	K	R	R	K	R	R	K	K
	150	A	A	A	A	V	A	A	A	A
	154	K	K	K	R	K	K	K	R	R
	166	K	K	K	R	K	K	K	K	K
	220	S	N	N	S	N	S	S	N	S
	235	I	I	V	V	I	I	I	V	V
	252	I	I	I	V	I	I	I	I	V
	411	D	D	D	E	D	D	D	D	D
	413	V	V	V	M	V	V	V	V	V
	433	D	N	D	D	D	D	D	D	D
	498	Q	Q	R	Q	Q	Q	Q	Q	Q
	500	V	V	I	V	I	V	V	I	V
	566	L	V	L	L	L	L	L	L	L
	577	I	V	I	I	V	I	I	I	I
	622	V	I	V	V	V	V	V	V	V
	660	D	D	D	E	D	D	D	D	D
	663	K	R	K	K	K	K	K	K	K
	736	F	Y	Y	Y	Y	F	F	Y	Y
	782	V	V	I	V	V	V	V	I	I
**protein**	**aa #**	**orange**	**green**	**cyan**	**navy**	**red**	**P**	**Wi61**	**IAL28**	**DS-1[e]**
**VP3**	21	L	L	L	L	I	L	L	L	T
	69	P	P	L	P	P	P	L	P	I
	98	I	V	V	V	V	I	V	V	V
	115	I/T	I	T	I	I	I	T	I	E
	116	D	D	E	D	N	D	E	D	D
	137	H	H	H	Q	H	H	H	H	H
	143	S/L	I	S	I	I	S	S	S	R
	155	I	I	V	I	I	I	V	V	I
	203	Y	H	H	H	H	Y	H	H	H
	277	H	Y	H	Y	Y	H	H	H	H
	281	I	I	I	I	V	I	I	I	I
	294	H	Y	H	Y	Y	H	H	H	Y
	301	D	D	D	D	N	D	D	D	D
	328	V	V	V	V	I	V	V	V	V
	346	K	G	K	G	G	K	K	K	G

	356	V	I	V	I	I	V	I	V	V
	364	N	S	S/G	S	N	N	S	S	D
	373	S/A	S	A	S	S	S	A	S	S
	405	V	I/V	V	V	I	V	V	V	I
	415	A/V	V	V	A	V	A	V	V	I
	420	T	T	N	T	T	T	N	T	N
	337	I	V	I	V	V	I	I	I	T
	452	A/V	T	A	T	M	A	A	A	T
	453	P	P/L	P	S	P	P	P	P	K
	502	N	N	N	N	S	N	N	N	G
	521	I	I	V	I	V	I	V	I	I
	531	M	I	M	I	M	M	M	M	I
	558	Y	D	Y	D	Y	Y	Y	Y	F
	625	A	A	T	A	A	A	T	T	S
	663	K	K	R	K	K	K	R	K	R
	683	G	G	E	G	R	G	E	E	N
	716	V	IV	I	V	V	V	V	V	V
	731	I	I	I	I	V	I	I	I	L
	733	D	D	D	D	N	D	D	D	D
	735	V	I	V	I	I	V	V	V	I
	748	T	M	T	M	M	T	T	M	M
	762	I	M/T	M	T	I	I	M	V	V
	767	A	A	A	A	V	A	A	A	I
	786	I	I	I	I	V	I	I	I	I
	807	T	T	T	I	T	T	T	T	T
	816	K	R	K	R	R	K	K	K	Y
**protein**	**aa#**	**orange**	**green**	**cyan**	**navy**	**red**	**P**	**Wi61**	**IAL28**	**DS-1[e]**
**VP6**	56	I	I	I	I	A	I	I	I	T
	80	T	N	N	N	N	T	T	N	T
	120	A	A	A	A	S	A	A	A	S
	130	N	D	D	D	D	N	N	D	D
	199	L	I	I	I	I	L	L	I	I
	217	A	V	A	A	A	A	A	A	V
	252	I	V	V	V	V	I	I	V	V
	291	S	S	S	L	S	S	S	S	S
**protein**	**aa #**	**orange**	**green**	**cyan**	**navy**	**red**	**P**	**Wi61**	**IAL28**	**DS-1[e]**
**NSP1**	10	H	Y	Y	Y	H	H	Y	Y	Q
	19	H	H	H	H	N	H	H	H	N
	20	T	V	T	A	T	T	T	A	A
	55	R	Q	R	R	S	R	R	R	Q
	68	Q	Q	Q	Q	R	Q	Q	Q	Q
	76	N/D	S/T	N	S	N	N	N	S	D
	93	D	N	D	N	D	D	D	N	S
	96	K	M	M	M	K	K	M	M	E
	103	E	N	D	D	E	E	D	N	N
	107	M	I	M	I	M	M	M	I	I
	110	Q	Q	Q	Q	H	Q	Q	Q	K
	111	K	R	K	R	K	K	K	K	K
	114	C	D	D	D	C/Y	C	D	D	H
	115	R	K	K	K	R	R	K	K	K
	117	I	I	I	M	I	I	I	I	A
	119	N	S	N	G	T	N	N	S	T
	130	M	M	M	I	M	M	M	M	L
	146	M	L	L	L	L	M	L	L	L

154	V	V/I	I	V	V	V	I	V	I
163	N	S	D	N	N	N	D	G	D
165	N	S	N	S	N	N	N	N	H
166	I	I	V	I	I	I	V	I	I
175	T	T	I	T	I	T	I	T	T
177	V	I	V	I	V	V	I	I	I
180	I	I	I	M	I	I	I	M	K
181	Y	Y	C	Y	Y	Y	C	Y	Y
188	D	N	D	N	D	D	D	N	S
189	V	I	V	I	V	V	V	V	I
192	A	N	T	N	T	A	T	N	D
199	A	V	I	V/I	A	A	I	L	L
200	S	V	T	A	S	S	T	A	T
117	S	S	S	S	N	S	S	S	T
119	Q	K	Q	K	Q	Q	Q	K	R
224	N	S	S	S	N	N	S	S	E
226	S	L	S	L	S	S	S	L	L
230	I	A	I	A	I	I	I	V	D
233	L	I	L	I	L	L	L	I	L
242	A	I	I	I	I	V	I	I	L
250	S	I	S	S	S	S	S	S	S
251	E	E	E	D	E	E	E	E	N
252	L	L	L	F	L	L	L	L	M
255	F	S	S	S	F	F	S	S	V
258	E	G	G	G	E	E	G	G	K
265	N	D	D	D	N	N	D	D	N
266	V	A	T	V	V	V	T	V	I
267	S	S	S	N	S	S	S	N	S
271	D	D	N	N	D	D	N	G	D
272	M	M	I	V	M	M	I	V	I
278	I	I	V	I	I	I	V	T	T
283	V	I	V	I	V	V	V	I	I
289	I	V	M	V	I	I	M	V	M
293	C	H	R	H	C	C	R	H	Y
296	K	K	Q	K	K	K	Q	K	K
297	M	I	I	I	M	M	I	I	I
301	L	H	L	H	L	L	L	Y	L
302	I	I	M	I	I	I	M	I	I
307	I/V	V	M	V	I	I	M	V	V
312	K	R	R	K	K	K	R	R	N
313	T	T	A	T	T	T	A	A	S
314	L/S	S	S	S	L	L	S	S	L
326	V	I	T/I	I	V	V	T	I	I
331	T	T	I	T	T	T	I	T	T
339	I	V	V	V	I	I	V	V	I
346	F	F	L	F	F	F	L	F	F
357	V	A	V	A	V	V	V	A	L
359	I	V	V	V	I	I	V	V	V
371	Y	H	C	Y	Y	Y	C	Y	S
372	V	I	V	I	V	V	V	I	I
373	E	E	E	K	K	E	E	E	K
374	D	D	N	D	D	D	N	N	D
375	V	V	I	V	V	V	I	V	V
377	N	D	D	D	N	N	D	D	N

	378	V	V	I	V	V	V	I	A	V
	381	D	D	N	D	D	D	N	D	E
	383	R	K	K	K	R	R	K	K	E
	386	T	T	T	I	T	T	T	T	M
	387	S	A	S	A	S	S	S	S	L
	388	I/V	V	V	V	I	I	V	V	V
	389	M	T	T	T	M	M	T	T	E
	391	D	N	N	N	D	D	N	N	N
	392	D	A	A	N	D	D	A	A	E
	394	K	K	N	K	K	K	N	K	N
	396	V	V	V	A	V	V	V	V	I
	412	I	V	V	V	I	I	V	V	I
	419	V	L	I	L	V	V	I	L	V
	422	H	Q	Q	Q	H	H	Q	Q	N
	424	I	I/V	I	V	I	I	I	V	I
	425	G	G	G	D	G	G	G	G	G
	434	E	N	N	D	E	E	N	N	S
	435	N	D	D	D	N	N	D	D	D
	436	V	I	V	I	V	V	V	I	V
	437	I	V	V	V	I	I	V	I	I
	438	T	I	T	I	T	T	T	I	L
	441	Q	K	Q/H	K	Q	Q	Q	Q	R
	459	V	T	T	T	V	V	T	T	T
	463	I	V	V	V	I	I	V	V	V
	470	H	C	C	C	Y	H	C	C	Y
	472	G	G	E	G	G	G	E	G	K
	473	L	V	P	V	S	L	P	M	D
	477	E	D	D	D	E	E	D	D	E
	484	I	V	V	V	I	I	V	V	I
**protein**	**aa #**	**orange**	**green**	**cyan**	**red**	**P**	**Wi61**	**IAL28**	**DS-1[e]**	
**NSP2**	24	S	N	N	S	S	N	S	N	
	48	V	V	I	V	V	I	I	I	
	58	K	K	K	R	K	K	K	K	
	64	N	S	S	N	N	S	N	N	
	75	P/S	I	V/A	P	P	V	P	S	
	82	I	V	V	I	I	V	V	A	
	91	I	I	L	I	I	L	I	T	
	93	V	I	V	V	V	V	V	V	
	135	A	S	S	A	A	S	S	S	
	175	K	K	E/D	K	K	E	K	K	
	200	I	V/A	V	I	I	V	V	S	
	202	V	I	V	V	V	V	I	I	
	248	N	S	I	N	N	I	N	N	
	255	T	I	I	T	T	I	I	L	
	293	M	M	I/T	M	M	I	M	I	
**protein**	**aa #**	**orange**	**green**	**cyan**	**navy**	**red**	**P**	**Wi61**	**IAL28**	**DS-1[e]**
**NSP3**	9	S	S	N	G	S	S	S	G	S
	65	I	I	V	M	I	I	I	M	A
	76	G	G	S	G	G	G	G	G	S
	78	A	A	A	V	A	A	A	V	S
	79	I	I	V	V	I	I	I	V	I
	104	T	T	M	I	I	T	I	I	L
	113	K	K	R	K	K	K	K	K	K
	141	K	K	R	R	K	K	K	R	K

	155	Y	Y	F	F	Y	Y	Y	F	F
	186	S/N	N	S	N	N	S	N	N	N
	191	T	T	N	N	T	T	T	N	N
	215	N	N	N	S	N	N	N	S	A
	225	N	N	N	S	N	N	N	S	N
	229	A	A	T	T/N	A	A	T	N	R
	233	G	G	N	N	S/G	G	G	N	S
	235	F	F	I	I	F	F	F	L	I
	237	S	S	L	S	S	S	S	S	S
	252	S/P	P	S	S	P	S	P	S	S
	255	V	V	I	I	V	V	V	I	I
	268	L	L	A	A	L	L	L	A	H
	278	I	I	L	L	I	I	I	L	F
	291	R	R	R	K	R	R	R	K	R
	295	M	M	M	M	I	M	M	M	M
	300	L	L	V	A	L	L	L	A	L
	301	K	K	R	R	K	K	K	R	Q
	307	Y	Y	Y	C	Y	Y	Y	C	Y
	308	A/V	A	T	T	A	A	T	T	T

protein	aa #	orange	green	cyan	navy	red	P	Wi61	IAL28	DS-1[e]
NSP4	70	Y	Y	C	Y	H	Y	C	C	Y
	72	I	I	I	I/V	M	I	I	I	I
	91	T	T	T	T	N	T	T	T	T
	129	R	R	R	R	S	R	R	S	R
	136	T	T	I	T	T	T	I	T	V
	137	R	K	K	R	R	R	K	K	R
	148	S/P	P	P	T	P	S	P	P	S
	161	S	S	S/N	S	N	S	S	S	N
	168	P	P	P	P	S	P	P	P	P
	169	S/T	S	L	S	S	P	P	P	P

protein	aa #	orange	green	cyan	red	P	Wi61	IAL28	DS-1[e]
NSP5	34	S	N	N	N	S	S	N	S
	38	I	V	V	V	I	I	V	I
	40	P	S	S	P	P	P	S	P
	42	A	I	I	I	A	V	I	A
	43	E	E	E	D	E	E	E	E
	105	L	M	M	M	L	L	M	M
	118	N	S	S	S	N	N	S	T
	123	I	V	V	V	I	I	V	I
	128	D	N	N	N	D	D	N	T
	129	H/N	H	H/Y	Q	H	H	H	S
	142	S	S	N	S	S	S	S	Y
	188	R	Q	Q	Q	R	Q	Q	Q

[a]numbering based on RRV VP7 (GenBank# AF295303)
[b]numbering based on RRV VP4 (GenBank# AY033150)
[c]numbering based on strain P (GenBank# EF583037-EF583037 and EF67598- EF67604)
[d]variation in the VP2 amino terminus (aa 1-47) not included in this table
[e]strain DS-1 represents genotype 2 for VP1-3, VP6, and NSP1-5
yellow filled boxes respresent novel amino acid residues not yet seen in sequenced RVs
