# Supplementary material for: Evolutionary Dynamics of Human Rotaviruses: Balancing Reassortment with Preferred Genome Constellations
Source: PLoS Pathog. 2009 Oct 23;5(10):e1000634. doi: 10.1371/journal.ppat.1000634 (PMC2760143; doi:10.1371/journal.ppat.1000634)
Supplement: Table S3 — Residues Defining Neutralization Domains of RRV VP7 (0.04 MB PDF) [file ppat.1000634.s003.pdf]

Table S3. Residues Defining Neutralization Domains of RRV VP7

Domain 7-1A		Domain 7-1B		Domain 7-2	
Align	Escape	Align	Escape	Align	Escape
T87	T87		Q201	K143	
E88		L208		Y144	
A90		D211	D211	D145	D145
T91	T91	T212		A146	
E92		A213	A213	T147	T147
N94	N94	T214		L148	L148
N96	N96	D238	D238	Q149	
S97	S97	V239		S189	
	W98	T240		S190	S190
K99	K99	T241		E217	E217
D100	D100	A242		T220	
	Q104			A221	
F118				K223	
K119					G264
E120				K291	K291
T122					
D123					
S126					
D130					

Numbering based on RRV sequence (AF295303)
Align: residues on the outer surface of the VP7 trimer that show G-type specific variation based on G1, G2, G3, G4, and G9
Escape: residues defined by neutralization escape mutants
