# Supplementary material for: Evolutionary Dynamics of Human Rotaviruses: Balancing Reassortment with Preferred Genome Constellations
Source: PLoS Pathog. 2009 Oct 23;5(10):e1000634. doi: 10.1371/journal.ppat.1000634 (PMC2760143; doi:10.1371/journal.ppat.1000634)
Supplement: Table S4 — Residues Defining Neutralization Domains of RRV VP8* (0.05 MB PDF) [file ppat.1000634.s004.pdf]

Table S4. Residues Defining Neutralization Domains of RRV VP8*

Domain 8-1		Domain 8-2		Domain 8-3		Domain 8-4	
Align	Escape	Align	Escape	Align	Escape	Align	Escape
N98			E180	N111		P86	
T99		N183	N183	T113			T87
	D100				S114		A88
R101				E115			A89
	T146				T116	G90	
	Q148			S118		L107	
N149				T124		C203	
	G150			Q125		D204	
Y152				E126			
S153				I128			
Q154				T129			
Y155				A131			
H170					N132		
N171					A133		
G172				S134			
K173					Q135		
	Y188				T136		
	S190			Q137			
T192				T161			
N193				P162			
	Y194						
D195							
S196							
N198							

Numbering based on RRV sequence (AF295303)
Align: residues on the outer surface of VP8* that show P-type specific variation based on P[3], P[4], P[6], and P[8]
Escape: residues defined by neutralization escape mutants
