# Supplementary material for: Evolutionary Dynamics of Human Rotaviruses: Balancing Reassortment with Preferred Genome Constellations
Source: PLoS Pathog. 2009 Oct 23;5(10):e1000634. doi: 10.1371/journal.ppat.1000634 (PMC2760143; doi:10.1371/journal.ppat.1000634)
Supplement: Table S5 — Residues Defining Neutralization Domains of RRV VP5* (0.04 MB PDF) [file ppat.1000634.s005.pdf]

Table S5. Residues Defining Neutralization Domains of RRV VP5*

Escape (Domain)	Align	
D384 (5-1)	A250	V353
S386 (5-1)	V255	Q360
A388 (5-1)	V256	I379
Q393 (5-1)	T272	T381
W394 (5-1)	A278	V396
T398 (5-1)	I281	S406
T440 (5-1)	S284	R429
R441 (5-1)	F296	T431
E434 (5-2)	P298	E433
K459 (5-3)	T304	S436
R429 (5-4)	E311	S438
T306 (5-5)	M323	V444
	D325	R446
	F328	A453
	V338	K459
		V464
		A465
		S476

Numbering based on RRV sequence (AF295303)
Escape: residues defined by neutralization escape mutants
Align: residues on the outer surface of VP5* that show
P-type specific variation based on P[3], P[4], P[6] and P[8]
