# Supplementary material for: Evolutionary Dynamics of Human Rotaviruses: Balancing Reassortment with Preferred Genome Constellations
Source: PLoS Pathog. 2009 Oct 23;5(10):e1000634. doi: 10.1371/journal.ppat.1000634 (PMC2760143; doi:10.1371/journal.ppat.1000634)
Supplement: Table S6 — Primers Used for RT-PCR and Sequencing (0.07 MB PDF) [file ppat.1000634.s006.pdf]

Table S6. Primers Used for RT-PCR and Sequencing

T01A01VP1A1FM	TGTAAAACGACGGCCAGTGGCTATTAAAGCTRTAC
T01A02VP1A1FM	TGTAAAACGACGGCCAGTGGCTATTAAAGCTRTAC
T01A03VP1A1BFM	TGTAAAACGACGGCCAGTGGCTATTAAAGCTRTACAATGG
T01A04VP1A499FM	TGTAAAACGACGGCCAGTAAAATTTATAARMGDAGAYTRG
T01A05VP1A646FM	TGTAAAACGACGGCCAGTTCWATWGARATGYTWATGTCRG
T01A06VP1A679FM	TGTAAAACGACGGCCAGTGAAGATTAYTTAATHGCRARAG
T01A07VP1A1105FM	TGTAAAACGACGGCCAGTTAYACDATGYTRATWAGAGATG
T01A08VP1A1228FM	TGTAAAACGACGGCCAGTGAATCMAGRCARCTHAAATTTGG
T01A09VP1A1283FM	TGTAAAACGACGGCCAGTTGCAYGTNATGGATGAYATBGC
T01A10VP1A1723FM	TGTAAAACGACGGCCAGTATGRAYTCATAYGTTCAAATACC
T01A11VP1A1775FM	TGTAAAACGACGGCCAGTATGGWGCTGTHGCWTCAGG
T01A12VP1A1903FM	TGTAAAACGACGGCCAGTGAYGGYGATGAYAAYTAYGC
T01B01VP1A2301FM	TGTAAAACGACGGCCAGTTACRTTYAARGTRTTTGAYTCAG
T01B02VP1A2405FM	TGTAAAACGACGGCCAGTCRGGAATAGCTGATGARATHGC
T01B03VP1A2620FM	TGTAAAACGACGGCCAGTAAATCAARYAAAATWACDATWAAYG
T01B04VP1A2851FM	TGTAAAACGACGGCCAGTGARGARYTRTATAAAGTWATWTC
T01B05VP2A1FM	TGTAAAACGACGGCCAGTGGCTATTAAAGGHTCAATGGC
T01B06VP2A1BFM	TGTAAAACGACGGCCAGTGGCTATTAAAGGHTCA
T01B07VP2A1CFM	TGTAAAACGACGGCCAGTGGCTATTAAAGGHTCAATG
T01B08VP2A443FM	TGTAAAACGACGGCCAGTAARYTRTTTAGAATATTTGAACC
T01B09VP2A596FM	TGTAAAACGACGGCCAGTCAARTDCTDAYWGAAATGCCRG
T01B10VP2A648FM	TGTAAAACGACGGCCAGTAAAAYAARAAYTCWAGRGATGC
T01B11VP2A1117FM	TGTAAAACGACGGCCAGTAGCDCARATHCARAARATGTC
T01B12VP2A1277FM	TGTAAAACGACGGCCAGTGTRACTACNAAYTATATGTC
T01C01VP2A1322FM	TGTAAAACGACGGCCAGTACHGTHRTRCCAAAYGATATG
T01C02VP2A1649FM	TGTAAAACGACGGCCAGTATGCCMRTTGATTATAARAGRTC
T01C03VP2A1952FM	TGTAAAACGACGGCCAGTGTRGCTATHATWACKGCNGCHAATAG
T01C04VP2A2169FM	TGTAAAACGACGGCCAGTGWGCHTCAGAYAAAATTGC
T01C05VP2A2225FM	TGTAAAACGACGGCCAGTGAAAGAGATGARATGTAYGG
T01C06VP3A1FM	TGTAAAACGACGGCCAGTGGCTATTAAAGC
T01C07VP3A2FM	TGTAAAACGACGGCCAGTGCTATTAAAGCARTAYYAG
T01C08VP3A22FM	TGTAAAACGACGGCCAGTAGTGYGTTTTACCTCTGATGG
T01C09VP3A398FM	TGTAAAACGACGGCCAGTTATYTAYTRCCWGGWTGGA
T01C10VP3A563FM	TGTAAAACGACGGCCAGTAARATYAARGARAGRATGAC
T01C11VP3A815FM	TGTAAAACGACGGCCAGTATYGGWCARTATAARAAYATG
T01C12VP3A908FM	TGTAAAACGACGGCCAGTTCAGCDCCHTCDTAYTGGA
T01D01VP3A1190FM	TGTAAAACGACGGCCAGTTGYGTTAAAWTRACHGCYATGG
T01D02VP3A1364FM	TGTAAAACGACGGCCAGTACWGAVAAYGTRTTYATAC
T01D03VP3A1805FM	TGTAAAACGACGGCCAGTTCRGGDTAYATWTTTAGRG
T01D04VP3A1889FM	TGTAAAACGACGGCCAGTTATAAYGCATTRATTTAYTAYAG
T01D05VP3A1985FM	TGTAAAACGACGGCCAGTARRTATTAYGAACAYGCWCC
T01D06VP4A1FM	TGTAAAACGACGGCCAGTGGCTATAAAATG
T01D07VP4A1BFM	TGTAAAACGACGGCCAGTGGCTATAAAATGGCTTC
T01D08VP4A1BFM	TGTAAAACGACGGCCAGTGCTATAAAATGGCTTC
T01D09VP4A388FM	TGTAAAACGACGGCCAGTGAAARTAARCARTTTAATGTRAG
T01D10VP4A539FM	TGTAAAACGACGGCCAGTTATGGACRTTTCATGGYGAAAC
T01D11VP4A1042FM	TGTAAAACGACGGCCAGTTAYGAAGTYATYAAAGARAATTC
T01D12VP4A1209FM	TGTAAAACGACGGCCAGTGAATGGWGGYGCHGTTTCG
T01E01VP4A1630FM	TGTAAAACGACGGCCAGTATTGATYTRACYAAATCAATGGC
T01E02VP4A1645FM	TGTAAAACGACGGCCAGTTCAATGGCRRCTAGYGTAATG
T01E03VP4A1705FM	TGTAAAACGACGGCCAGTGAAATGACTARYTCATTRTCAG

T01E04NSP1A9FM	TGTAAAACGACGGCCAGTTTTATGAAAAGTCTTG
T01E05NSP1A9BFM	TGTAAAACGACGGCCAGTTTTATGAAAAGTCTTGTG
T01E06NSP1A17FM	TGTAAAACGACGGCCAGTAAGTCTTGTGKAAGCC
T01E07NSP1A278FM	TGTAAAACGACGGCCAGTAGAATGMGRACWTTYARRAATG
T01E08NSP1A497FM	TGTAAAACGACGGCCAGTTATTAYRTATTTGGDTAYTATG
T01E09NSP1A899FM	TGTAAAACGACGGCCAGTAGATGTARANTRTTYAC
T01E10NSP1A995FM	TGTAAAACGACGGCCAGTGTDCAYAATTGYAARTGGTG
T01E11NSP1A995FM	TGTAAAACGACGGCCAGTGTDCAYAATTGYAARTGGTG
T01E12VP6A1FM	TGTAAAACGACGGCCAGTGGCTTTWAAACGAAGTCTTC
T01F01VP6A1FM	TGTAAAACGACGGCCAGTGGCTTTWAAACGAAGTCTTC
T01F02VP6A1BFM	TGTAAAACGACGGCCAGTGGCTTTWAAACGAAGTC
T01F03VP6A405FM	TGTAAAACGACGGCCAGTARAGRATAAAYTTTRATAATTCRTC
T01F04VP6A485FM	TGTAAAACGACGGCCAGTTTTCATAAACCWAAYATHTTYCC
T01F05VP6A554FM	TGTAAAACGACGGCCAGTAAYTTRATGGGDACNATGTGG
T01F06VP6A642FM	TGTAAAACGACGGCCAGTAAHAYHCARCARTTTGARCA
T01F07VP6A719FM	TGTAAAACGACGGCCAGTGAAAGATTYAGTTTTCCAAGAGT
T01F08NSP3A1FM	TGTAAAACGACGGCCAGTGGCWTTTAATGCTTTTCAG
T01F09NSP3A1BFM	TGTAAAACGACGGCCAGTGGCTTTTAATGCTTTTCAGTGG
T01F10NSP3A1BFM	TGTAAAACGACGGCCAGTGGCTTTTAATGCTTTTCAGTGG
T01F11NSP3A270FM	TGTAAAACGACGGCCAGTTNAGRAAYAGAAATTGGATG
T01F12NSP3A356FM	TGTAAAACGACGGCCAGTAAAGGAATYGAYCAAAARATGAG
T01G01NSP3A452FM	TGTAAAACGACGGCCAGTATGAARGANAAAATWGAACGYGG
T01G02NSP2A1FM	TGTAAAACGACGGCCAGTGGCTTTTAAMGCGTCTCAG
T01G03NSP2A1FM	TGTAAAACGACGGCCAGTGGCTTTTAAMGCGTCTCAG
T01G04NSP2A1BFM	TGTAAAACGACGGCCAGTGGCTTTTWAMGCGTCTCAGTC
T01G05NSP2A359FM	TGTAAAACGACGGCCAGTGTAGTTTCYGTWAGACATYTDG
T01G06NSP2A395FM	TGTAAAACGACGGCCAGTAGRARAGARAAYCATCARGAYG
T01G07NSP2A524FM	TGTAAAACGACGGCCAGTCARAATRCWGCWTTTACWATGTGG
T01G08VP7A1FM	TGTAAAACGACGGCCAGTGGCTTTAAAAGMGAG
T01G09VP7A1BFM	TGTAAAACGACGGCCAGTGGCTTTAAAARMGAGAATTTCC
T01G10VP7A1CFM	TGTAAAACGACGGCCAGTGGCTTTAAAAGAGAGAATTTCC
T01G11VP7A364FM	TGTAAAACGACGGCCAGTTTTHTWACWAAAGGDTGGCC
T01G12VP7A535FM	TGTAAAACGACGGCCAGTTGGYTRTGTAAYCCDATGG
T01H01VP7A592FM	TGTAAAACGACGGCCAGTAATAARTGGATWTCRATGGG
T01H02NSP4A1FM	TGTAAAACGACGGCCAGTGGCTTTTAAAAGTTCTGTTCCG
T01H03NSP4A398FM	TGTAAAACGACGGCCAGTTRACTACDCGTGAAATTGARC
T01H04NSP4A1FM	TGTAAAACGACGGCCAGTGGCTTTTAAAAGTTCTGTTCCG
T01H05NSP4A3FM	TGTAAAACGACGGCCAGTCTTTTAAAAGTTCTGTTCCGAG
T01H06NSP5A1FM	TGTAAAACGACGGCCAGTGGCTTTWAAAGCGCTACAGTG
T01H07NSP5A1FM	TGTAAAACGACGGCCAGTGGCTTTWAAAGCGCTACAGTG
T01H08NSP5A1BFM	TGTAAAACGACGGCCAGTGGCTTTWAAAGCGCTACAG
T01A01VP1A623RM	CAGGAAACAGCTATGACCTARTAYGGTTTATCYTTCATYAC
T01A02VP1A734RM	CAGGAAACAGCTATGACCGTYGATCTATTWGARTATGA
T01A03VP1A806RM	CAGGAAACAGCTATGACCGTAATRAAYGTWCCRTTDATATC
T01A04VP1A1226RM	CAGGAAACAGCTATGACCCCATTYGAHGCTGAHGACATTG
T01A05VP1A1435RM	CAGGAAACAGCTATGACCGYGCWATAAARTAYTCATAYGG
T01A06VP1A1373RM	CAGGAAACAGCTATGACCCTHCKHCCTARYGGWATTGG
T01A07VP1A1813RM	CAGGAAACAGCTATGACCCTGCYTTHGTYTGYTTYTC
T01A08VP1A1918RM	CAGGAAACAGCTATGACCARTTRTCATCDCCRTCRACTC
T01A09VP1A2021RM	CAGGAAACAGCTATGACCGCTTTRACTTTAGCRTTCATWC
T01A10VP1A2426RM	CAGGAAACAGCTATGACCGMWATYTCATCAGCTATTCCYG
T01A11VP1A2548RM	CAGGAAACAGCTATGACCGTGCATAYGAATTYAAYTTYGC
T01A12VP1A2586RM	CAGGAAACAGCTATGACCHGCYGATATYTGYGCACG
T01B01VP1A3033RM	CAGGAAACAGCTATGACCYTGATAACATCCRTAATTRATWG

T01B02VP1A3300RM	CAGGAAACAGCTATGACCTCACATCTAAGCRCTCTAATC
T01B03VP1A3302RM	CAGGAAACAGCTATGACCGGTCACATCTAAGCRCTC
T01B04VP1A3302RM	CAGGAAACAGCTATGACCGGTCACATCTAAGCRCTC
T01B05VP2A538RM	CAGGAAACAGCTATGACCYTTYTTYAAYTTCCAATACCATC
T01B06VP2A657RM	CAGGAAACAGCTATGACCTTYTTRTTTTCDACDGCCATATC
T01B07VP2A768RM	CAGGAAACAGCTATGACCCTCATWTCYGCRATRAAYCTTC
T01B08VP2A1214RM	CAGGAAACAGCTATGACCTDGCTGCTTGTGAATTTATRCC
T01B09VP2A1443RM	CAGGAAACAGCTATGACCGGRTCNCCRTTYCTATARTG
T01B10VP2A1419RM	CAGGAAACAGCTATGACCCKTTGCATTCCRAAYGCTGG
T01B11VP2A1798RM	CAGGAAACAGCTATGACCRGTTTGAACATGYTGCATRTTC
T01B12VP2A2079RM	CAGGAAACAGCTATGACCCTRTACATTTGRTCRTCYGG
T01C01VP2A2246RM	CAGGAAACAGCTATGACCAWCCRTACATYTCATCYCTYTC
T01C02VP2A2412RM	CAGGAAACAGCTATGACCATWAGYGATATDACTGATGAATC
T01C03VP2A2733RM	CAGGAAACAGCTATGACCCTCCACARTGGGGTTGRCG
T01C04VP2A2759RM	CAGGAAACAGCTATGACCCCCAYTGTGGAGATATGACC
T01C05VP2A2759RM	CAGGAAACAGCTATGACCCCCAYTGTGGAGATATGACC
T01C06VP3A498RM	CAGGAAACAGCTATGACCTCHGTAGCHGCRTTYTGAC
T01C07VP3A637RM	CAGGAAACAGCTATGACCAAATAAYTTRTCTCTRAAHAC
T01C08VP3A705RM	CAGGAAACAGCTATGACCAAWGTRAACATDGAYTCATTYC
T01C09VP3A980RM	CAGGAAACAGCTATGACCCYARTGGRTCCCAYGTYTC
T01C10VP3A1344RM	CAGGAAACAGCTATGACCGMRTAWAAYACWCCTTTTGG
T01C11VP3A1587RM	CAGGAAACAGCTATGACCGTTGGYARAAAHGTCCAATC
T01C12VP3A1698RM	CAGGAAACAGCTATGACCATRAAYAARTGATTATTWCCRG
T01D01VP3A1932RM	CAGGAAACAGCTATGACCAGRTCAAAHGHRTARTTATATC
T01D02VP3A2133RM	CAGGAAACAGCTATGACCTCRGCRTADGTTATRCTATAHAC
T01D03VP3A2579RM	CAGGAAACAGCTATGACCACYAGTGTGTTAAGTTTYTAGC
T01D04VP3A2591RM	CAGGAAACAGCTATGACCGGTCACATCRTGACYAGTGTG
T01D05VP3A2591RM	CAGGAAACAGCTATGACCGGTCACATCRTGACYAGTGTG
T01D06VP4A536RM	CAGGAAACAGCTATGACCCTWCCAYCATATTTYRATATTCC
T01D07VP4A662RM	CAGGAAACAGCTATGACCTTRGAYTCTTGDGACCTTGG
T01D08VP4A761RM	CAGGAAACAGCTATGACCGCTCTYKTATAYTGTATVGATC
T01D09VP4A1152RM	CAGGAAACAGCTATGACCCACTGARTTYAAATTAGCTGC
T01D10VP4A1313RM	CAGGAAACAGCTATGACCACTGTCAAACTAAATCTAAAYCG
T01D11VP4A1768RM	CAGGAAACAGCTATGACCTCGATCTRAYRGAAACGYTTC
T01D12VP4A1976RM	CAGGAAACAGCTATGACCTTYCCAATTTGAGTRGACATATC
T01E01VP4A2370RM	CAGGAAACAGCTATGACCGTCACATCCWMMMTAGCRTTC
T01E02VP4A2371RM	CAGGAAACAGCTATGACCGGTCACATCCTCAATAGC
T01E03VP4A2371BRM	CAGGAAACAGCTATGACCGGTCACATCCWMMMTAGC
T01E04NSP1A417RM	CAGGAAACAGCTATGACCTCRTTTCTACAYTTAYGYTG
T01E05NSP1A468RM	CAGGAAACAGCTATGACCGAYTGTAAHGTWATTGGC
T01E06NSP1A660RM	CAGGAAACAGCTATGACCTATCTRARTGCATAYTCTTGYTG
T01E07NSP1A1014RM	CAGGAAACAGCTATGACCCACCATTTRCAATTRTGHAC
T01E08NSP1A1141RM	CAGGAAACAGCTATGACCTTCYTSYGAHGARCAATGTCC
T01E09NSP1A1570RM	CAGGAAACAGCTATGACCCGCTACTCTAGTGCAGRGAG
T01E10NSP1A1586RM	CAGGAAACAGCTATGACCTTTWWTGCTGCC
T01E11NSP1A1596RM	CAGGAAACAGCTATGACCGGTCACATTTTATGCTG
T01E12VP6A510RM	CAGGAAACAGCTATGACCTAWGGRAAWATRTTWGGTTTATG
T01F01VP6A609RM	CAGGAAACAGCTATGACCAATCCRGCNACYTGAATYTCTG
T01F02VP6A755RM	CAGGAAACAGCTATGACCCWGCTGAATTAATHACTCTTGG
T01F03VP6A1137RM	CAGGAAACAGCTATGACCTCWGTCCAATTCATRCCTGG
T01F04VP6A1167RM	CAGGAAACAGCTATGACCTCYCTRGATGGTGAATARTTAG
T01F05VP6A1364RM	CAGGAAACAGCTATGACCGGTCACATCCTCTCAC
T01F06VP6A1364RM	CAGGAAACAGCTATGACCGGTCACATCCTCTCAC
T01F07VP6A1364BRM	CAGGAAACAGCTATGACCGGTCACATCCTCTCACT

T01F08NSP3A535RM	CAGGAAACAGCTATGACCCCAATCWATNGTRTCAAYTTCC
T01F09NSP3A552RM	CAGGAAACAGCTATGACCTGWTCATAWCKWGATTTCCAATC
T01F10NSP3A664RM	CAGGAAACAGCTATGACCAATRACRTTYTGAAGAGAGTWC
T01F11NSP3A1065RM	CAGGAAACAGCTATGACCTATAGCCATTTAGGTTTTTGAC
T01F12NSP3A1081RM	CAGGAAACAGCTATGACCGGTCACATAACGCC
T01G01NSP3A1081RM	CAGGAAACAGCTATGACCGGTCACATAACGCC
T01G02NSP2A553RM	CAGGAAACAGCTATGACCYARTYTCCACATWGTAAAWGC
T01G03NSP2A642RM	CAGGAAACAGCTATGACCTCTGARABTGGTTTRTCTTC
T01G04NSP2A728RM	CAGGAAACAGCTATGACCCTCTRTARTGACCTTTACCATG
T01G05NSP2A1053RM	CAGGAAACAGCTATGACCATAAGCGCTTTCWATTCTYRC
T01G06NSP2A1059RM	CAGGAAACAGCTATGACCGGTCACATAAGCGCTTTC
T01G07NSP2A1059RM	CAGGAAACAGCTATGACCGGTCACATAAGCGCTTTC
T01G08VP7A616RM	CAGGAAACAGCTATGACCATGNTCCCATYGAWATCC
T01G09VP7A741RM	CAGGAAACAGCTATGACCATCWACRACRTCMRTWATCAC
T01G10VP7A656RM	CAGGAAACAGCTATGACCAATGTTTGYGTATTYARNGGACA
T01G11VP7A1063RM	CAGGAAACAGCTATGACCGGTCACATCRWACAATTC
T01G12VP7A1063RM	CAGGAAACAGCTATGACCGGTCACATCRWACAATTC
T01H01VP7A1063RM	CAGGAAACAGCTATGACCGGTCACATCRWACAATTC
T01H02NSP4A580RM	CAGGAAACAGCTATGACCCTYACATNGMTGCAGTCACTTC
T01H03NSP4A762RM	CAGGAAACAGCTATGACCGGTCACRYTAAGACCRTTCC
T01H04NSP4A449RM	CAGGAAACAGCTATGACCCCRTTCCTTCCATTAACGTCC
T01H05NSP4A762RM	CAGGAAACAGCTATGACCGGTCACRYTAAGACCRTTCC
T01H06NSP5A673RM	CAGGAAACAGCTATGACCGGTCACAAAACGGGAG
T01H07NSP5A673BRM	CAGGAAACAGCTATGACCGGTCACAAAACGGGAGTGG
T01H08NSP5A649RM	CAGGAAACAGCTATGACCTCCCTAGTGTGCTCTCAGG
